# Theta-Defensins to Counter COVID-19 as Furin Inhibitors: *In Silico* Efficiency Prediction and Novel Compound Design

**DOI:** 10.1155/2022/9735626

**Published:** 2022-02-09

**Authors:** Manica Negahdaripour, Mohammad Reza Rahbar, Zahra Mosalanejad, Ahmad Gholami

**Affiliations:** ^1^Pharmaceutical Sciences Research Center, Shiraz University of Medical Sciences, Shiraz, Iran; ^2^Department of Pharmaceutical Biotechnology, School of Pharmacy, Shiraz University of Medical Sciences, Shiraz, Iran; ^3^Biotechnology Research Center, Shiraz University of Medical Sciences, Shiraz, Iran

## Abstract

Coronavirus disease 2019 (COVID-19), caused by severe acute respiratory syndrome coronavirus 2 (SARS-CoV-2), was characterized as a pandemic by the World Health Organization (WHO) in Dec. 2019. SARS-CoV-2 binds to the cell membrane through spike proteins on its surface and infects the cell. Furin, a host-cell enzyme, possesses a binding site for the spike protein. Thus, molecules that block furin could potentially be a therapeutic solution. Defensins are antimicrobial peptides that can hypothetically inhibit furin because of their arginine-rich structure. Theta-defensins, a subclass of defensins, have attracted attention as drug candidates due to their small size, unique structure, and involvement in several defense mechanisms. Theta-defensins could be a potential treatment for COVID-19 through furin inhibition and an anti-inflammatory mechanism. Note that inflammatory events are a significant and deadly condition that could happen at the later stages of COVID-19 infection. Here, the potential of theta-defensins against SARS-CoV-2 infection was investigated through *in silico* approaches. Based on docking analysis results, theta-defensins can function as furin inhibitors. Additionally, a novel candidate peptide against COVID-19 with optimal properties regarding antigenicity, stability, electrostatic potential, and binding strength was proposed. Further *in vitro*/*in vivo* investigations could verify the efficiency of the designed novel peptide.

## 1. Introduction

The new coronavirus, called “severe acute respiratory syndrome coronavirus 2” (SARS-CoV-2), was detected in China in Dec. 2019 for the first time as the agent of the coronavirus disease 2019 (COVID-19) outbreak [1], which was announced as a pandemic by the World Health Organization (WHO) in March 2020. It has caused more than 276 million confirmed cases and more than five million deaths globally as of December 2021 [2]. Numerous clinical trials were started to investigate the efficacy and safety of various drugs such as hydroxychloroquine, lopinavir/ritonavir, corticosteroids, and remdesivir for this disease, and guidelines are being updated continually [3]. Combinations of drugs with different mechanisms might be more effective [4]. Fortunately, several vaccines have been developed and are being used, but antiviral drugs are still needed to save the lives of infected people. Moreover, various mutations on the SARS-CoV-2 genome indicate the need for drugs besides vaccines. Treatment approaches are divided into two broad categories according to the targets: (1) inhibition of enzymes involved in viral pathogenesis and (2) inhibition of lung injury that happens due to inflammation by immune system modulation [5].

Coronaviruses have spike glycoproteins on their surface, which bind to the cell membrane and infect the cell [6]. A large ectodomain, a single-pass transmembrane anchor, and a short C-terminal intracellular tail are the components found on the spike glycoprotein [7]. The ectodomain consists of a receptor-binding unit, called S1, and a membrane-fusion unit, named S2 [8]. For the virus entry into host cells, binding of S1 to a specific cell surface receptor through its receptor-binding domain (RBD) is the first step, followed by S2 fusion with the host cell, which finally leads to infusion of the viral genome into host cells [9].

Specific cell enzymes such as furin, which has a site on the spike protein, can facilitate the entry process of the viral genome [8]. Furin is a host-cell enzyme classified as a family member of proprotein convertases. A total of nine members of this family have been identified in humans [10]. Furin is a proprotein convertase subtilisin/Kexin type 3, also termed PACE (paired basic amino acid cleaving enzyme), because it cleaves basic amino acid motifs. Furin regulates the activity of various mammalian, bacterial, and viral proteins. Therefore, it can be considered a target for treating different infectious and noninfectious diseases [11]. This host-cell enzyme is regarded as necessary in the viral maturation process and seems to be involved in SARS-CoV-2 pathogenesis and probably viral transfer among humans [12]. Thus, furin protein blockade is suggested as a therapeutic strategy against SARS-COV-2 [13].

Several peptidyl and nonpeptidyl furin inhibitors have been developed in the last decade. Some have shown promising results in mouse models [14], but only a few have entered human clinical trials for cancer treatment [11, 15]. The promising results of peptide-based furin inhibitors in treating viral infections have been reviewed [16].

Defensins are arginine-rich antimicrobial peptides, which can hypothetically inhibit furin since it was proven that poly-arginine-derived molecules could inhibit the activity of furin protein [17]. Plants, animals, and fungi express defensins as a defense tool [18, 19], regarded as essential for the host response to infections. Defensins have antimicrobial effects against Gram-positive and Gram-negative bacteria, fungi, parasitic protozoa, and enveloped and nonenveloped viruses [18, 20]. They target several different stages of the virus life cycle [21], such as viral replication [22], and can bind to viral or host proteins [23, 24]. Defensin can also modulate innate and adaptive immune responses [25].

Defensins are usually cationic with multiple cysteines in their structure [18], which secure a beta-sheet core structure through three conserved intramolecular disulfide bonds [26]. There are glycine and positively charged amino acids (arginine and lysine) in the defensin structure, as well as aliphatic hydrophobic residues that form a hydrophobic core [27].

Three families of mammalian defensins are identified based on their structure, including alpha-, beta-, and theta-defensins (TDs) [28]. TDs contain several arginine residues and cyclic peptides in their structure. They are expressed in some animal species, such as Old World monkeys, and cannot be found in human cells because of a premature stop codon [29]. Synthetic humanized TDs could have a therapeutic effect on bacterial and viral lung infections [30] and have anti-infective and anti-inflammatory properties [31]. These peptides act against viruses (such as HIV and influenza) by various mechanisms such as direct interaction with the virus [32, 33], protease inhibition [34], and suppression of proinflammatory responses [35]. A rhesus type of TD, Rhesus theta-defensin-1 (RTD-1), protected mice against SARS infection, a possible mechanism of which was the immunomodulatory activity of RTD-1 [36]. TDs have attracted attention as anti-infective drug candidates due to their small size, unique structures, and involvement in several defense mechanisms [29].

Bioinformatics tools help study different compounds and predict their features in less time and lower costs than the lab experiments, giving the insight to find or design more efficient structures [37, 38]. Computational approaches could be of great value given the importance and urgency of finding therapeutics molecules for COVID-19. This study investigates the potential of TD against SARS-CoV-2 through an *in silico* analysis of the TD structure and delves into the nature of TD-furin interaction by docking approaches. Finally, a candidate novel peptide against COVID-19 with furin inhibition and anti-inflammatory activity would be proposed.

## 2. Computational Methods

### 2.1. Data Retrieval

The keywords “human furin” and “theta defensin” were explored to obtain their sequence and structural data from the UniProt Knowledgebase (UniProtKB) [39] at https://www.uniprot.org/, Protein Data Bank (PDB) [40] at https://www.rcsb.org/, and PepBDB [41] at http://huanglab.phys.hust.edu.cn/pepbdb/. The reference sequence of furin (UniProt ID: P09958) was searched against the clusters of coordinates archived in the PDBFlex database [42] (http://pdbflex.org/) to limit the effect of structural redundancies. Each cluster in the PDBFlex database comprises structures with at least 95% sequence identity.

To find any peptide-reactive furin interactions, querying the amino acid sequence of furin was also conducted in PepBDB. PepBDB is a database containing information about biological peptide-mediated protein interactions of the peptides in the PDB with a length of up to 50 residues.

### 2.2. Sequence Comparison

Structural alignment of TD sequences was performed by the T-Coffee expresso algorithm at http://tcoffee.org/ [43] for sequence comparison. The duplicated sequences were removed before alignment by the duplicate finder java standalone application. The alignment file was visualized and explored by the Alvis alignment visualizer [44].

### 2.3. TD Consensus Patterns

The conserved patterns on TD sequences were discovered using the PRATT algorithm [45] as provided at https://web.expasy.org/pratt/. The PRATT tool enables finding conserved patterns in a group of protein sequences.

### 2.4. Antigenicity

The antigenicity of each defensin was assessed by the VaxiJen server [46] at http://www.ddg-pharmfac.net/. VaxiJen helps to search for protective antigens through an alignment-independent method, which performs the prediction about the physicochemical properties of proteins.

### 2.5. Electrostatic Potential

The electrostatic potential of each defensin peptide and furin was measured based on the electrostatic surface grid calculated according to Coulomb's law [47]. First, the molecular surfaces were displayed by the UCSF Chimera program [48], which could be used for molecular modeling, visualization, and analysis. The surface-enclosed residues were used to calculate the electrostatic potential. The distance-dependent dielectric was selected to vary in proportion to the distance from each charge. The dielectric constant was set at 4. The distance from the surface vertex was 1.4; the protonation state was based on a local H-bonding environment. The surface coloring was performed by the “Coulombic Surface Coloring” function of the UCSF Chimera program [48]. The molecular surface coloring is performed in this method by the potential values. It can be used for structures with or without explicit hydrogens and could produce a grid of potential values.

### 2.6. Investigating the Furin Properties

The properties of furin active sites were assessed by the CAVER Analyst version 2 standalone software [49]. The approach was initiated by defining the active sites extracted from the databases. The volume data was based on the Voss Volume Voxelator (3V) algorithm [50], as provided at http://3vee.molmovdb.org/. The 3V encompasses several programs for estimating volumes in protein files.

The properties of the furin cleft were assessed by the CavityPlus server [51] at http://www.pkumdl.cn/. It helps to detect and analyze the functions of protein cavities using 3D structural information of proteins.

### 2.7. Docking

The binding properties of furin and the available TDs were defined by applying a hybrid approach strategy (template-based modeling into free docking with default parameters) by HDOCK [52] (http://hdock.phys.hust.edu.cn/).

A peptide-based docking approach was employed on all peptide sequences separately by GalaxyPepDock, available at http://galaxy.seoklab.org. The server employs a combination of information on similar interactions found in the structural databases and model building through energy-based optimization [53].

### 2.8. Network View of Peptide-Furin Interactions

The networks of the interaction of each available TD with furin were extracted from the docked structures. First, the TD peptide structure was selected within the protein complex; then, the network of interactions between the selected residues and other residues in the complex was extracted by using RINanalyzer [54], as embedded in Cytoscape 3.7.2 [55]. The RINanalyzer plugin of Cytoscape allows creating an interaction network of amino acid residues directly from the structure by connecting the UCSF Chimera with Cytoscape. The residue interaction networks included hydrogen bonds, combined edges, contacts, clashes, backbone connectivity, and distances. The extracted networks were visualized and explored by using the Cytoscape software tool.

### 2.9. Centrality Analysis of Interaction Networks

Centrality analysis included defining the average shortest path length (ASPL) change under the removal of the individual nodes (nodes in the networks are representative of residues in the protein structure) [56]. The approach was made by the RINspector application [57] of Cytoscape. A *z*-score was calculated for each node based on modifying the average shortest path length upon removal of each node. The central residues, which are the residues that play an essential role in the communication within the interaction network, were assigned by a significant *z*-score (*z*-score > 2).

### 2.10. Nature-Inspired Peptide Design

#### 2.10.1. Construct Properties and Modeling

The tertiary structure of the construct was built by the *de novo* method at https://bioserv.rpbs.univ-paris-diderot.fr/services/PEP-FOLD/ by the PEP-FOLD server [58] and the Modeller software [59] using the graphical user interface available in Chimera. The structure prediction was performed using a homology modeling approach. The quality of the predicted structures was evaluated by MolProbity [60] available at http://molprobity.biochem.duke.edu/.

The Disulfide by Design, provided at http://cptweb.cpt.wayne.edu/, was employed to examine the disulfide bonds in the predicted structure.

The construct dynamics were determined and visualized by sampling conformations using the normal analysis mode of the Dynamut server [61] (http://biosig.unimelb.edu.au/dynamut/). The C-alpha force field was selected for standard mode analysis calculations, which was derived from fitting to the Amber94 all-atom potential.

The designed construct was subjected to the aforementioned evaluations, including antigenicity, stability, electrostatic potential, and binding strength.

## 3. Results

### 3.1. Data Mining

Due to the presence of several solved structures in PDB for the 794-amino acid reference sequence of furin (UniProtKB ID: P09955), the amino acid sequence of furin was obtained using the archived cluster of structures (PDBFlex database). The results included a range of clusters at a similarity level of 49.01 (cluster master: 6F9M) to 99.36 (cluster master: 6HZA). 6HZA was comprised of 43 members, among which the best resolution was related to the X-ray diffraction solution of furin under the accession number of 5JXG and resolution of 1.80 Å.

### 3.2. Furin Properties

The reactive residues, active site, and active cleft of furin were obtained using databases and literature. The available UniProtKB data on the furin reference sequence is summarized in Table S1 of Supplementary File 1. D153, H194, and S368 were introduced as active sites based on the charge relay system (Prosite rule). Furthermore, the interactive residues in the furin-peptide complexes, available in PepDB, revealed that approximately 30 residues of furin are involved in the interaction of this enzyme with short peptides. The interactive peptides all included arginine with a length of 5-7 residues (Supplementary File 1, Table S2). These data provided a reference to confirm the docking results and helped define the interactive residues in the docking process.

#### 3.2.1. Furin Cleft

**Figure 1:**
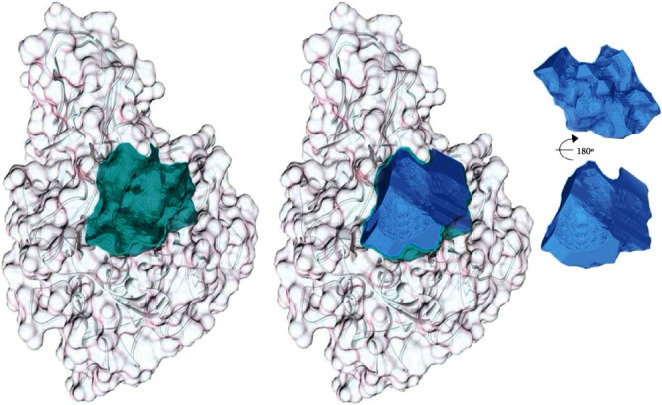
The cartoon representation of furin and its active cleft. The left panel is the furin structure with its active cleft identified by green color. The active cleft is separated in the other panels, as shown in the right panel from the forward and reverse view. The active cleft is presented as a vacant area in the furin structure in the middle image.

All cavities on the structure surface were identified to measure the volume and area of the furin cleft. At least nine cavities were recognized on the surface of furin (PDB entry 5JXG, Figure S1). A large spherical cavity with a volume of 1968 Å^3^, an area of 1371 Å^2^, and sphericity of 0.8 presents the active cleft of the enzyme. The route to the active site also passed through this relatively large cavity (Figure 1). The maximal pKd of the binding site was 10.13, which revealed the druggability of this cavity.

**Figure 2:**
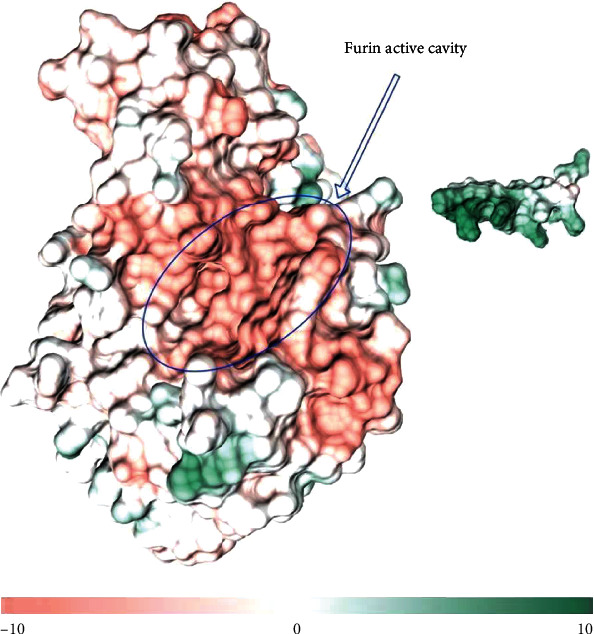
The electrostatic potential of the furin surface. The left panel is the furin structure in which the surfaces are colored by a color gradient from salmon-red to green; the active cleft is specified by a blue oval shape. The right panel is the structure of the designed construct colored by a color gradient as well. The low electrostatic potential of the furin active cleft and the high electrostatic potential of the peptide construct are evident in the figure.

The electrostatic measurement of the surface area showed the extremely negative electrostatic potential of the active cleft (-28) (Figure 2). As shown in Figure 2, the active cavity and its surroundings comprised a negative electrostatic potential, whereas the other areas showed a medium potential. The high potentials were scattered through small surfaces (green regions in Figure 2).

### 3.3. TDs

#### 3.3.1. Sequence Analysis

All defensins used in the present study were short (approximately 18-residue long) peptides. The existence of several arginines and cysteine residues in the sequences of all defensins is mentionable. The arginine and cysteines are both the fundamental elements that appear in the sequence pattern. All sequences obey a consensus pattern, namely, R-C-[ILV]-C-*x*-R-*x*(2)-C-R-C-[IV]-C, where *x* is any amino acid; this pattern served as a template for designing a novel construct.

The stability and antigenicity of each peptide were based on the physicochemical properties and amino acid composition of the sequences.  Table 1 shows the sequence stability and antigenicity of each peptide in which instability indices lower than 40 are considered stable, and a lower number offers more stability. Thus, the most stable TD was 2ATG followed by 2LZ1, while other peptides were indicated to be unstable. However, these two peptides were presented with high antigenicity. The antigenicity threshold is 0.4, and the fewer antigenic peptides were 2M2H, 2M2X, 2M2G, 1HVZ, and 2LYE, respectively. Our designed construct had a low antigenicity with close-to-threshold stability.

#### 3.3.2. Structural Exploration

The list of TDs used in this study is as follows. The PDB IDs of TDs are 1HVZ, 2LYE, 2LZI, 2M1P, 2M2G, 2M2H, 2M2S, 2M2X, 2M2Y, 2M77, 2M78, and 2M79. These theta-defensins include both natural and synthetic molecules. Since all these structures are determined by nuclear magnetic resonance spectroscopy and contain several trajectories, the model for further analyses was selected based on the full validation report deposited in PDB. The medoid structure was selected. The medoid structure, which is representative of all trajectories [62, 63], is the model that is the most similar to all other models.

#### 3.3.3. The Convexity Indices

A convexity index was assigned for each residue to define the protruded side chains of the amino acids of TDs. The most protruded side chains were attributed to arginine (Supplementary Data 1, Table S3; Supplementary Figure S2).

#### 3.3.4. The Electrostatic Potential

A positive electrostatic complement peptide would engage the highly negative furin cleft. To account for this, along with calculating the furin electrostatic potential, similar values were calculated for each peptide. The results showed that all defensins were highly favorable in electrostatic potential (Supplementary Figure S3). This would explain the binding property of these peptides to the furin cleft. Moreover, the peptides' calculated volume and surface area revealed the appropriate size of these peptides to occupy the furin cleft (Table 2).

#### 3.3.5. Furin-Peptide Docking

Two docking approaches were performed to delve into the nature of theta-defensin-furin interaction. The first was based on the structures, and the second was on the peptide sequence and structure of furin. The structure-based method resulted in ten different orientations for each peptide structure and furin structure. Considering the docking scores, it can be observed that the minimal energies were not necessarily related to the correct orientation (Supplementary Data 1, Table S4). Our primary criterion defines the correct orientation (the engagement of the peptide structure in the furin active cleft). The minimal energetic interaction of correct orientation belonged to the 2M1P structure (-271.05).

### 3.4. Essential Residues in Protein Complexes

A centrality analysis outlined the essential residues involved in the peptide-furin complex. The *z*-score values (significant *z*-score: >2) of centrality reveal a notable role for arginine residues in each complex (Table 3 and Supplementary Figure S4). Instead, the cysteines were not attributed to significant *z*-scores. This emphasized the critical role of arginine residues in the peptide-protein interaction. In contrast, cysteines are critical in maintaining the structure of the peptides.

### 3.5. Rational Designing of the Construct

The data obtained from the previous sections provide a guideline for designing a novel peptide, which will be referred to as “construct” hereafter. The criteria for designing the construct are achieving a peptide with higher stability, lower antigenicity, and higher electrostatic potential than natural TDs. The sequence pattern extracted from the library of TDs provided a scaffold for rational designing of the novel construct. In the designing plan, the construct sequence was considered 18 to 20 sites. A close view of the pattern showed that these TDs are a tandem repeat of shorter sequences: ~9 residues in which the arginine residues are protruded from two side loops that connect a central beta-ladder. The three cysteine pairs are coupled face to face in the beta-ladder to form three disulfide bonds.

**Figure 3:**
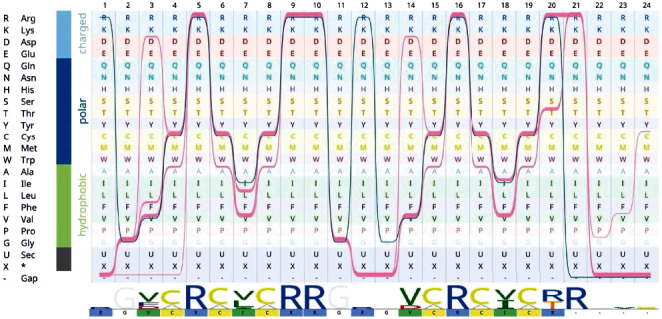
Multiple sequence alignment of theta-defensins was used in the present study. The alignment was visualized by sequence bundles. The sequence of the designed construct was also included in the alignment. Each line represents a peptide sequence that passed through the amino acid matrix introducing the peptide residue of the related site. Each sequence in the alignment block can be identified by following each line. When the bundles stack on an amino acid, a conserved site is introduced. The arrangement of amino acids was based on physicochemical properties specified at the left. At the bottom, the sequence logo also shows the conservancy of each side; the size of fonts is proportional to the entropy in the alignment column. Below the sequence logo, the sequence of the designed construct is presented by color codes.

The pattern remained constant, and the variable residues were selected from the options, which were limited to the existing residues identified in the sequence alignment (Figure 3).

The proper residues were selected based on centrality *z*-scores; the residues of the significant *z*-score were selected within other options. Moreover, the scarcity of the residues in the antibody-antigen interface was followed to lower the antigenicity of the construct.

### 3.6. Construct Structure

**Figure 4:**
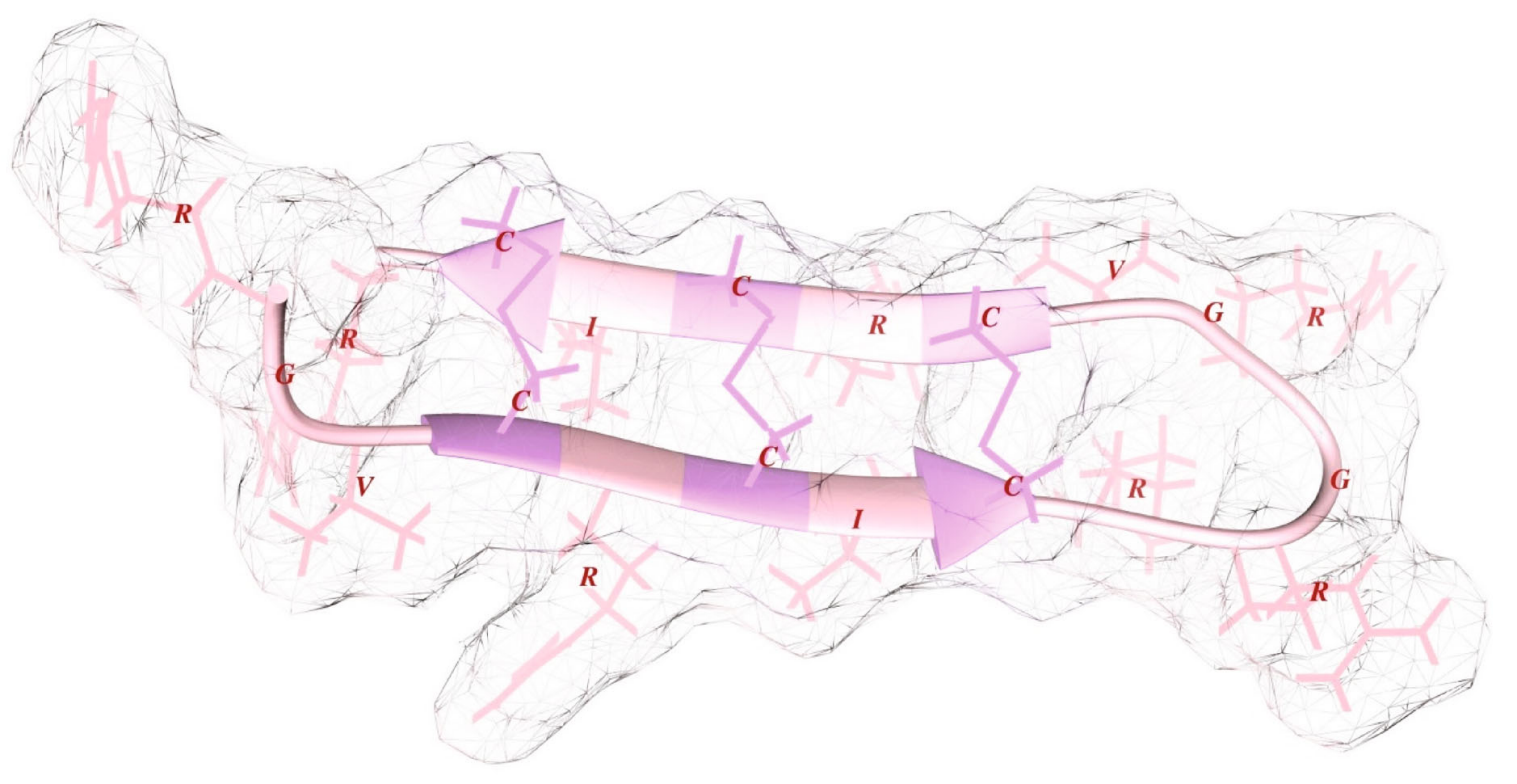
Cartoon presentation of the designed construct. The ribbon shows the secondary structure elements of the construct. Cysteine residues and disulfide bonds are illustrated by hot pink color. All side chains are presented by thick sticks. Residues are specified by a one-letter code. The surface of the molecule is presented by transparent mesh lines.

The *de novo* modeling method and homology modeling approach revealed slightly different structures, yet the homology modeling approach yielded a more satisfactory structure (Supplementary Data 1, Table S5). In the homology modeling approach, two structures of 2LYE and 5NIZ served as templates with 88.9 percent sequence identity. The resulting modeled structures comprised the ZDOPE score of 0.62 to 1.7. The best structure (with the lower ZDOPE value) was selected for further investigations. In the modeled structure, the three expected disulfide bonds are appropriately located with *χ*_3_ angles of 101.32, 116.66, and 117.09; the energy of 0.73, 2.20, and 1.48 kcal/mol; and *Σ* B-factor of 143.08, 158.14, and 429.42 (the *χ*_3_ angle is typically -87° or +97° ± 30). The predicted structure of the construct with its residue side chains and disulfide bonds is presented in Figure 4.

In Figure 2, the right panel also indicates the too high electrostatic potential of the construct (+10).

**Figure 5:**
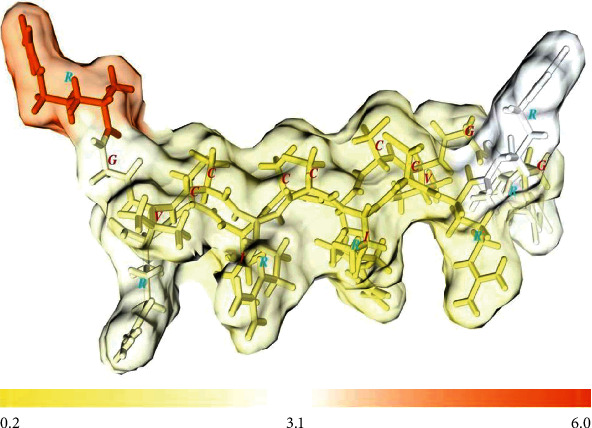
Backbone trace of the construct. The molecule was colored based on convexity (protrusion) indices in a gradient from yellow to red. Residues, bonds, and side chains are presented by thick sticks and specified by one-letter codes. The color key is at the bottom right.

The convexity index list of atoms in the construct structure showed the high protrusion value of arginine side chains (Figure 5 and Supplementary Data 1, Table S3); the highest sum of all-atom convexity index belonged to Arg10 (41.95), and the most protruded atoms were hydrogens (6.19).

To explore the properties of construct-furin interaction, the peptide construct was docked to furin. The minimal energy within the top ten predictions represented the correct orientation of the construct (Table 4). The template for docking was furin in the complex with a competitive inhibitor (meta-guanidinomethyl-phenyl acetyl-Arg-Val-Arg-(amidomethyl) benzamidine) under the PDB accession number of 4OMC [64].

**Figure 6:**
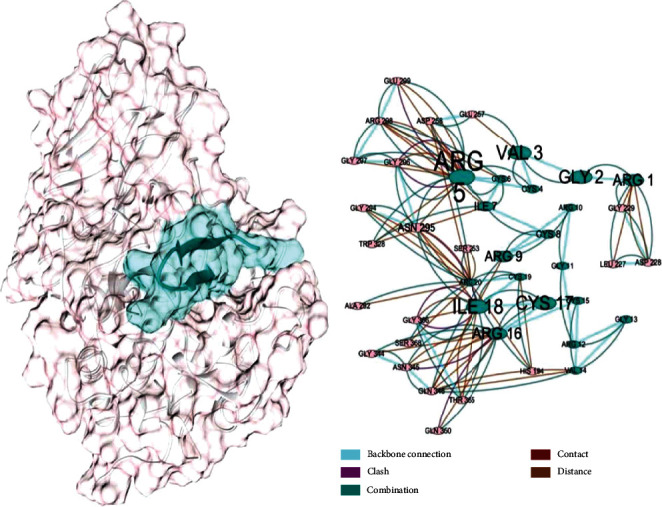
Schematic presentation of the construct-furin complex and interaction network. (a) The cartoon representation of the peptide construct located in the furin active cleft. Molecular surfaces of the designed construct and furin are colored in green and light pink, respectively; the molecular surfaces are at 80 percent transparency. (b) The molecular interaction network extracted from the construct-furin complex. It depicts different interactions between construct residues and furin residues. Each node in the network is representative of a protein residue. The residue names are shown by three-letter codes. The size of nodes and their name fonts are proportional to the *z*-score of centrality; the edges show different types of interactions introduced by color keys at the bottom right. The node colors are synchronized by the left structure image.

The interaction network of the construct-furin complex showed that the selected residues had significant *z*-scores (Figure 6, Table 3).

**Figure 7:**
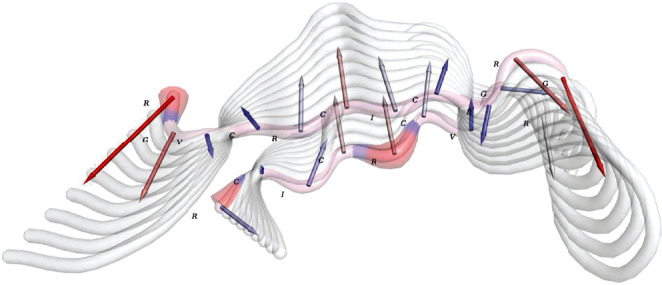
Schematic illustration of the construct dynamics. The backbone of the construct is presented by rounded ribbons. The trajectories, vectors, and molecular fluctuations are merged to depict an informative illustration. The arrows show the vectors colored by blue to red gradient representing low to high levels, respectively. The trajectories are presented by multiple white rounded ribbons consistent with vectors. Thick blue to red regions are molecular fluctuations. Construct residues are defined by one-letter codes.

To examine the flexibility of the construct, the molecular fluctuation was predicted. The vectors, trajectories, and fluctuations were merged to represent more flexible regions and the movement vectors (Figure 7). The very ends of the molecule comprised the highest level of fluctuations; the trajectories in Figure 7 evidenced the highly fluctuating (therefore, more flexible) arginine residues. Additionally, arginine and isoleucine residues fluctuated in the opposite direction of the two ends at the middle point.

## 4. Discussion

Given the role of furin in SARS-CoV-2 pathogenicity and the fact that inhibition of host enzymes could be a better approach than viral enzymes, furin is believed to be an important therapeutic target in COVID-19 management. Some findings support this theory. For instance, genetic variations of furin cause different binding affinities toward viral spike glycoprotein and result in various infectivity rates in humans [65]. Additionally, the plasma level of furin was found to increase in patients with coronary artery disease and COVID-19, which may be the reason for their poor clinical prognosis [66].

TDs are considered furin inhibitors and thus could be employed in the treatment of some viral infections, namely, COVID-19. In this study, the structure of TDs and their interaction with furin were examined in detail to verify the abovementioned notion and help design a similar construct with more beneficial features.

The low electrostatic potential of the furin active cleft and high electrostatic potential of the TDs, as well as the appropriate size of these peptides for insertion into the furin cleft, are evidence of a strong interaction between furin and TDs, which could lead to furin inhibition by TDs.

The observed pattern of TDs provided a clue for modifying their structure to obtain an improved TD with better binding property, which can engage with the furin cleft. Mining the data of the available structures provided clues for designing a novel peptide that would better occupy the furin cleft and interact more strongly besides possessing less immunogenicity, more stability, and more flexibility. The original template for designing the construct was the basic sequence pattern consensus within the available TDs. Further modifications were applied for meeting the criteria, as mentioned earlier. The values higher than six suggested that the binding site might be a suitable target for drug designing.

The results showed that peptide modifications led to a novel construct with more stability and hydrophilicity as well as a smaller size than natural TD. The physicochemical properties of the novel TD suggested it as an appropriate furin inhibitor or, in other words, a more robust binder.

Arginine residues have an essential role in TD-furin interaction. This result corresponds to the hypothesis that defensins, as arginine-rich molecules, can inhibit furin. On the other hand, cysteines are critical in maintaining the structure of TD. The arginine residues are protruded from two side loops that connect a central beta-ladder. The three cysteine pairs are coupled face to face in the beta-ladder to form three disulfide bonds. Arginine and isoleucine residue fluctuations cause the flexibility of the molecule. The nonpolar amino acids help the hydrophilic-lipophilic balance.

According to Table 1, most of the TD peptides represented antigenicity. Some structures, such as the 2M2H structure, had low antigenicity but were not stable enough. The minimal energy of interaction of correct orientation is related to the 2M1P structure (-271.05). Nevertheless, its antigenic properties and stability were not suitable. Therefore, an optimum state should be considered. The designed peptide (construct) had higher stability, lower antigenicity, and higher electrostatic potential. Generally, in docking, the minimal energy within the top ten predictions indicates the correct orientation of the construct. Following this criterion helped in designing a better construct.

Constrained peptides such as TDs are good choices for drug development [67]. Smaller cyclic peptides with the same activity of TD have been developed before [68, 69], which possessed enhanced antiviral properties and immunosuppressive activity [70].

TDs, as a group of cyclic peptides, have high stability and low toxicity. Moreover, cyclic structures usually show good affinity and selectivity because of the increased surface area and conformational rigidity [68].

In animal models, TDs have demonstrated minimal immunogenicity, low toxicity [35, 71], and thermal and proteolytic stability [72]. TDs are *β*-hairpin antimicrobial peptides, but their amphiphilicity is low, which causes their low hemolytic activity [73]. Compared with alpha- and beta-defensins, TD activities are relatively insensitive to salt, divalent cations, and serum [74]. For instance, a hybrid peptide of TD and human beta-defensin-1 showed salt-resistant property [75]. TDs are a type of lectin and can bind to carbohydrate-containing surface molecules, for example, when protecting against HIV-1 infection [76]. Aminoglycosides produce functional TDs in humans [77]. Therefore, they could be used for COVID-19 treatment [78].

One primary reason for COVID-19 severity and mortality is inflammatory cytokine induction at the late stages of the disease, known as cytokine storm or cytokine release syndrome (CRS), which may even lead to organ failure [79]. The organ damage in SARS-CoV-2-infected people seems immune-mediated, similar to autoimmune diseases [80]. Thus, identifying the stage of disease progression and timing of interventions is essential. At the early stages of the disease, it is best to reduce the number of viruses, while hyperinflammation should be controlled at more advanced stages [81, 82]. Thus, limiting the inflammatory responses could be as essential as preventing virus replication [83, 84] and have a life-saving role in COVID-19 patients [82]. It seems that TD can be helpful in both stages. TD has shown anti-inflammatory activity *in vitro* and *in vivo* by inhibiting proinflammatory cytokines' release. In this regard, TD activated the phosphatidylinositol 3-kinase (PI3K)/protein kinase B (AKT) pathway and suppressed proinflammatory signals in immune-stimulated cells. RTD-1 immunomodulation activity was identified in the TLR (Toll-like receptors) and TNF (tumor necrosis factor) pathways via suppressing NF-*κ*B (nuclear factor kappa-light-chain-enhancer of activated B cells) activation and expression as well as cytokine release [85–87]. In SARS-infected mice treated with RTD1, decreased levels of proinflammatory cytokines and increased survival were observed [35, 36].

Some studies showed that antiviral peptides could also be candidates for COVID-19 treatment [88–90], mainly through fusion/entry inhibition [89]. Their antiviral activity could be enhanced through conjugation with conventional antiviral drugs [90]. In this regard, several peptides were suggested for the treatment of COVID-19, such as protegrin-1, LL-37 (cathelicidin), beta-defensin 1 [91], and P9R, which is a defensin-like peptide [92]. Another defensin, human intestinal defensin-5, inhibited SARS-CoV-2 entry [93], as confirmed by the luciferase assay [94]. Alpha-defensins may potentially treat COVID-19 as well [95]. Mouse beta-defensin-4 has also shown antiviral activity *in vitro* and *in vivo* against SARS-CoV [96].

Presently, two clinical trials are recruiting patients to study the efficacy of defensins in COVID-19, including (1) a phase II clinical trial on a TD ([97] and (2) a phase II trial on brilacidin [98], a small-molecule human defensin mimetic. The primary mechanism of this molecule is disrupting the viral integrity and entry. Brilacidin has shown a synergistic effect with remdesivir *in vitro* [99].

Furin inhibitors provoke a lower probability of resistance induction because they do not target the virus [100]. However, furin inhibition might cause toxicity and side effects [101]. Some reports of triggering thrombosis in a mouse model, as the possible adverse effects of defensins, especially alpha-defensin, were reported. Additionally, some defensins promoted viral and bacterial infections in certain biological conditions. Hence, it is vital to consider their probable side effects [101, 102]. To reduce their adverse events, pulmonary delivery systems using nanoformulation to deliver antimicrobial peptides [103] could be an option.

## 5. Conclusion

Antiviral drugs for COVID-19 are in demand to save the lives of infected people, especially considering various mutations of the SARS-CoV-2 genome. Targeting the host-cell enzymes such as furin can cause less viral resistance. Despite some possible side effects, the advantages of short-term furin inhibition could outweigh [16].

According to our *in silico* results, TDs can act as furin inhibitors presented by the low electrostatic potential of furin active cleft and high electrostatic potential of the TDs, as well as the appropriate size of TDs for insertion into the furin cleft. Arginine residues have an essential role in TD*-*furin interaction. This relates to the notion that defensins, as arginine*-*rich molecules, can inhibit furin. Therefore, TDs could be a potential treatment for COVID-19 by furin inhibition and anti-inflammatory mechanisms. Furthermore, TDs have appropriate properties naturally, such as high stability and low toxicity—however, natural TDs have either high antigenicity or low stability in our analyses. Thus, a novel peptide with optimal antigenicity, stability, electrostatic potential, and binding strength was designed. However, further studies, including *in vitro* and *in vivo* experiments, are needed to confirm these results. Additionally, the proposed method could be employed for developing other novel proteins and peptides.

## Figures and Tables

**Table 1:** The antigenicity and stability of theta-defensins and the designed construct.

Peptide	Sequence	Instability index^∗^	Antigenicity^∗∗^
2LYE	GVCRCVCRRGVCRCVCRR	59.99	0.43
2M2Y	RCVCRRGVCRCVCRRGVC	55.28	0.65
2M1P	GVCRAVCRRGVCRAVCRR	68.37	0.87
2M2G	GVARCVCRRGVCRCVARR	59.99	0.38
2M2H	GVARCVARRGVARCVARR	59.99	0.09
2M2X	GVARAVARRGVARAVARR	68.37	0.31
1HVZ	GFCRCLCRRGVCRCICTR	65.36	0.42
2M77	GDCRCLCRRGVCRCICTR	65.36	0.76
2M78	GFCRCLCRRGDCRCICTR	65.36	0.98
2M79	GDCRCLCRRGDCRCICTR	65.36	1.31
2ATG	RRICRCICGRGICRCICG	31.83	1
2LZ1	GICRCICGRRICRCICGR	36.55	1.05
Construct	RGVCRCICRRGRGVCRCICRRG	46.18	0.37

^∗^The instability indices lower than 40 are considered stable. ^∗∗^The threshold of antigenicity is 0.4.

**Table 2:** The volume and surface data of theta-defensins.

Peptide	Volume A°3	Surface A°2
1HVZ	1966	1338
2YLF	1981	1405
2LZI	1887	1354
2M1P	1968	1371
2M2G	1939	1366
2M2H	1985	1352
2M2S	1981	1400
2M2X	1979	1389
2M77	1876	1386
2M78	1964	1360
2M79	1894	1341
2YLE	1946	1421
Construct	2178	1678

**Table 3:** The centrality score of different residues of theta-defensins in the peptide-furin complex. The columns are the residues related to the heading peptide.

2M78	2M79	2M77	2M2Y	2M2X	2M2S	2M2H	2M2G	2M1P	2LZI	2LYF	1HVZ	Site	Average	Pattern	Construct sequence	Designed construct
—	—	—	—	—	—	—	—	—	—	—	—	0.00	—		R	1.47
-0.45	-0.23	-0.33	0.85	-0.19	0.24	-0.54	-0.39	-0.60	-0.37	-0.63	-0.63	1	-0.27	gr	G	1.88
3.95	0.25	1.37	-0.21	-0.05	0.00	1.06	-0.67	-0.29	-0.37	0.84	0.84	2	0.56	vidf	V	2.10
-0.30	1.18	-0.38	3.68	-0.42	1.15	-0.39	-0.50	-0.23	0.90	-0.21	-0.21	3	0.36	c	C	0.17
3.86	1.87	2.31	1.33	1.13	1.86	5.03	-0.34	1.22	1.57	2.33	2.33	4	2.04	R	R	2.83
0.31	2.66	0.03	1.05	-0.18	-0.15	0.68	-0.33	1.24	-0.52	0.25	0.25	5	0.44	C	C	-0.61
0.85	1.03	4.63	0.11	-0.26	3.14	1.77	-0.34	3.41	1.13	0.03	0.03	6	1.29	ILV	I	0.65
-0.56	0.22	-0.74	-0.49	-0.35	2.86	0.04	-0.37	-0.61	-0.16	-0.72	-0.72	7	-0.13	C	C	0.38
2.31	2.87	0.59	-0.51	-0.33	-0.66	1.21	2.77	-0.54	-0.90	-0.46	-0.46	8	0.49	X	R	1.17
—	—	—	—	—	—	—	—	—	—	—	—		—		R	-0.47
—	—	—	—	—	—	—	—	—	—	—	—		—		G	-0.58
-0.27	-0.81	1.10	0.29	-0.34	1.72	-0.58	-0.07	2.48	1.67	0.57	0.57	9	0.53	R	R	-0.41
-0.28	-0.23	-0.12	-0.78	0.08	0.03	-0.28	-0.54	-0.66	-1.01	-0.39	-0.39	1	-0.38	X	G	-0.61
-0.34	-0.34	-0.33	0.35	-0.09	-0.28	0.03	0.39	-0.47	-1.01	1.59	1.59	11	0.09	X	V	-0.43
-0.57	-0.01	0.49	1.09	-0.21	0.37	-0.47	0.58	0.18	0.16	2.00	2.00	12	0.47	C	C	-0.73
-0.28	0.71	0.32	2.55	-0.33	-0.41	0.11	2.97	1.72	2.13	0.13	0.13	13	0.81	R	R	1.46
0.54	1.24	1.26	1.93	0.61	1.11	-0.36	0.66	-0.18	2.16	3.77	3.77	14	1.38	C	C	2.02
-0.14	0.99	-0.18	-0.44	0.94	-0.65	-0.19	0.08	1.41	-0.46	-0.71	-0.71	15	0.00	IV	I	2.20
-0.02	0.53	0.01	-0.84	-0.31	-0.65	-0.61	-0.54	0.39	1.01	0.05	0.05	16	-0.08	C	C	-0.55
-0.18	-0.09	-0.67	-0.49	1.35	0.13	-0.41	-0.19	-0.81	-0.81	-0.58	-0.58	17	-0.28	t	R	-0.46
-0.11		-0.35	-0.25	0.80	0.24	0.24	-0.37	2.52	1.30	1.18	1.18	18	0.58	r		

^∗^Significant *z*-score is defined as >2.

**Table 4:** Summary of construct-furin docking scores and ligand (construct) root mean square deviation (RMSD). The top ten orientations of the ligand in the peptide-furin complex are ranked from the best score (minimum energy) to the worst, respectively.

Rank	1	2	3	4	5	6	7	8	9	10
Docking score	-263.2	-230.51	-230.16	-230.06	-224.93	-221.9	-215.8	-215.5	-214.39	-214.27
Ligand RMSD (Å)	66.01	55.96	65.24	62.42	60.49	52.01	68.52	65.05	64.76	71.14

## Data Availability

All data generated or analyzed during this study are included in this published article and its Supplementary Information files.

## Supplementary Materials

Supplementary Materials Supplementary Data 1. Figure S1: cartoon representation of furin cavities. Figure S2: the convexity index for each residue of theta defensins. Figure S3: defensin positive electrostatic potential. Figure S4: a centrality analysis of the important residues involved in the peptide-furin complex. Supplementary Data 2. Table S1: the available UniProtKB data on the furin reference sequence. Table S2: the interactive residues in furin-peptide complexes (available in PepDB). Table S3: the convexity index for each residue of theta defensins. The most protruded side chain is arginine. Table S4: furin-peptide docking, the structure-based method, resulted in ten different orientations for each peptide structure and furin structure. Table S5: designing a novel peptide, which is referred to as “construct.” The criteria for designing the construct are achieving a peptide with higher stability, lower antigenicity, and higher electrostatic potential.

## References

[1] Hashemi S. A., Golab Behbahan N. G., Bahrani S. (2021). Ultra-sensitive viral glycoprotein detection NanoSystem toward accurate tracing SARS-CoV-2 in biological/non-biological media. *Biosensors and Bioelectronics*.

[2] https://covid19.who.int.

[3] Negahdaripour M. (2020). The rise and fall in therapeutic candidates for COVID-19. *Iranian Journal of Medical Sciences*.

[4] Tarighi P., Eftekhari S., Chizari M., Sabernavaei M., Jafari D., Mirzabeigi P. (2021). A review of potential suggested drugs for coronavirus disease (COVID-19) treatment. *European Journal of Pharmacology*.

[5] Wang R., Stephen P., Tao Y., Zhang W., Lin S.-X. (2021). Human endeavor for anti-SARS-CoV-2 pharmacotherapy: a major strategy to fight the pandemic. *Biomedicine & Pharmacotherapy*.

[6] Mousavi S. M., Hashemi S. A., Parvin N. (2020). Recent biotechnological approaches for treatment of novel COVID-19: from bench to clinical trial. *Drug Metabolism Reviews*.

[7] Li F. (2016). Structure, function, and evolution of coronavirus spike proteins. *Annual review of virology*.

[8] Owji H., Negahdaripour M., Hajighahramani N. (2020). Immunotherapeutic approaches to curtail COVID-19. *International Immunopharmacology*.

[9] Sheybani Z., Heydari Dokoohaki M., Negahdaripour M. (2021). The interactions of folate with the enzyme furin: a computational study. *RSC Advances*.

[10] Seidah N. G., Prat A. (2012). The biology and therapeutic targeting of the proprotein convertases. *Nature Reviews Drug Discovery*.

[11] Braun E., Sauter D. (2019). Furin-mediated protein processing in infectious diseases and cancer. *Clinical & Translational Immunology*.

[12] Alsibai K. D. (2020). Expression of angiotensin-converting enzyme 2 and protease in COVID-19 patients: a potential role of cellular FURIN in the pathogenesis of SARS-CoV-2. *Medical Hypotheses*.

[13] Rabaan A. A., al-Ahmed S. H., Haque S. (2020). SARS-CoV-2, SARS-CoV, and MERS-COV: a comparative overview. *Le Infezioni in Medicina*.

[14] Yakala G. K., Cabrera-Fuentes H. A., Crespo-Avilan G. E. (2019). FURIN inhibition reduces vascular remodeling and atherosclerotic lesion progression in mice. *Arteriosclerosis, Thrombosis, and Vascular Biology*.

[15] Oh J., Barve M., Matthews C. M. (2016). Phase II study of Vigil® DNA engineered immunotherapy as maintenance in advanced stage ovarian cancer. *Gynecologic Oncology*.

[16] Couture F., Kwiatkowska A., Dory Y. L., Day R. (2015). Therapeutic uses of furin and its inhibitors: a patent review. *Expert Opinion on Therapeutic Patents*.

[17] Cameron A., Appel J., Houghten R. A., Lindberg I. (2000). Polyarginines are potent furin inhibitors. *Journal of Biological Chemistry*.

[18] Ganz T. (2003). Defensins: antimicrobial peptides of innate immunity. *Nature Reviews Immunology*.

[19] Negahdaripour M., Owji H., Eslami M. (2019). Selected application of peptide molecules as pharmaceutical agents and in cosmeceuticals. *Expert Opinion on Biological Therapy*.

[20] Boulanger N., Lowenberger C., Volf P. (2004). Characterization of a defensin from the sand fly Phlebotomus duboscqi induced by challenge with bacteria or the protozoan parasite Leishmania major. *Infection and Immunity*.

[21] Brice D. C., Diamond G. (2020). Antiviral activities of human host defense peptides. *Current Medicinal Chemistry*.

[22] Pace B. T., Lackner A. A., Porter E., Pahar B. (2017). The role of defensins in HIV pathogenesis. *Mediators of Inflammation*.

[23] Demirkhanyan L. H., Marin M., Padilla-Parra S. (2012). Multifaceted mechanisms of HIV-1 entry inhibition by human *α*-defensin. *Journal of Biological Chemistry*.

[24] Nguyen E. K., Nemerow G. R., Smith J. G. (2010). Direct evidence from single-cell analysis that human *α*-defensins block adenovirus uncoating to neutralize infection. *Journal of Virology*.

[25] Holly M. K., Diaz K., Smith J. G. (2017). Defensins in viral infection and pathogenesis. *Annual Review of Virology*.

[26] Lehrer R. I., Bevins C. L., Ganz T. (2005). Defensins and other antimicrobial peptides and proteins. *Mucosal Immunology*.

[27] Rose G. D., Geselowitz A. R., Lesser G. J., Lee R. H., Zehfus M. H. (1985). Hydrophobicity of amino acid residues in globular proteins. *Science*.

[28] Selsted M. E. (2004). *θ*-Defensins: cyclic antimicrobial peptides produced by binary ligation of truncated *α*-defensins. *Current Protein and Peptide Science*.

[29] Lehrer R. I., Cole A. M., Selsted M. E. (2012). *θ*-Defensins: cyclic peptides with endless potential. *Journal of Biological Chemistry*.

[30] Tecle T., Tripathi S., Hartshorn K. L. (2010). Review: defensins and cathelicidins in lung immunity. *Innate Immunity*.

[31] Hazlett L., Wu M. (2011). Defensins in innate immunity. *Cell and Tissue Research*.

[32] Lehrer R. I. (2004). Primate defensins. *Nature Reviews Microbiology*.

[33] Penberthy W. T., Chari S., Cole A. L., Cole A. M. (2011). Retrocyclins and their activity against HIV-1. *Cellular and Molecular Life Sciences*.

[34] Rothan H. A., Han H. C., Ramasamy T. S., Othman S., Rahman N. A., Yusof R. (2012). Inhibition of dengue NS2B-NS3 protease and viral replication in Vero cells by recombinant retrocyclin-1. *BMC Infectious Diseases*.

[35] Schaal J. B., Tran D., Tran P. (2012). Rhesus macaque theta defensins suppress inflammatory cytokines and enhance survival in mouse models of bacteremic sepsis. *PLoS One*.

[36] Wohlford-Lenane C. L., Meyerholz D. K., Perlman S. (2009). Rhesus theta-defensin prevents death in a mouse model of severe acute respiratory syndrome coronavirus pulmonary disease. *Journal of Virology*.

[37] Gholami A., Shahin S., Mohkam M., Nezafat N., Ghasemi Y. (2015). Cloning, characterization and bioinformatics analysis of novel cytosine deaminase from *Escherichia coli* AGH09. *International Journal of Peptide Research and Therapeutics*.

[38] Hajighahramani N., Eslami M., Negahdaripour M. (2019). Computational design of a chimeric epitope-based vaccine to protect against *Staphylococcus aureus* infections. *Molecular and Cellular Probes*.

[39] Renaux A. (2018). UniProt: the universal protein knowledgebase. *Nucleic Acids Research*.

[40] Protein Data Bank (2019). The single global archive for 3D macromolecular structure data. *Nucleic Acids Research*.

[41] Wen Z., He J., Tao H., Huang S.-Y. (2019). PepBDB: a comprehensive structural database of biological peptide–protein interactions. *Bioinformatics*.

[42] Hrabe T., Li Z., Sedova M., Rotkiewicz P., Jaroszewski L., Godzik A. (2016). PDBFlex: exploring flexibility in protein structures. *Nucleic Acids Research*.

[43] Armougom F., Moretti S., Poirot O. (2006). Expresso: automatic incorporation of structural information in multiple sequence alignments using 3D-coffee. *Nucleic Acids Research*.

[44] Schwarz R. F., Tamuri A. U., Kultys M. (2016). ALVIS: interactive non-aggregative visualization and explorative analysis of multiple sequence alignments. *Nucleic Acids Research*.

[45] Jonassen I., Collins J. F., Higgins D. G. (1995). Finding flexible patterns in unaligned protein sequences. *Protein Science*.

[46] Doytchinova I. A., Flower D. R. (2007). VaxiJen: a server for prediction of protective antigens, tumour antigens and subunit vaccines. *BMC Bioinformatics*.

[47] Murray J. S., Politzer P. (2011). The electrostatic potential: an overview. *Wiley Interdisciplinary Reviews: Computational Molecular Science*.

[48] Pettersen E. F., Goddard T. D., Huang C. C. (2004). UCSF Chimera—a visualization system for exploratory research and analysis. *Journal of Computational Chemistry*.

[49] Jurcik A., Bednar D., Byska J. (2018). CAVER Analyst 2.0: analysis and visualization of channels and tunnels in protein structures and molecular dynamics trajectories. *Bioinformatics*.

[50] Voss N. R., Gerstein M. (2010). 3V: cavity, channel and cleft volume calculator and extractor. *Nucleic Acids Research*.

[51] Xu Y., Wang S., Hu Q. (2018). CavityPlus: a web server for protein cavity detection with pharmacophore modelling, allosteric site identification and covalent ligand binding ability prediction. *Nucleic Acids Research*.

[52] Yan Y., Tao H., He J., Huang S.-Y. (2020). The HDOCK server for integrated protein-protein docking. *Nature Protocols*.

[53] Lee H., Seok C. (2017). Template-based prediction of protein-peptide interactions by using GalaxyPepDock. *Modeling Peptide-Protein Interactions*.

[54] Doncheva N. T., Klein K., Domingues F. S., Albrecht M. (2011). Analyzing and visualizing residue networks of protein structures. *Trends in Biochemical Sciences*.

[55] Shannon P., Markiel A., Ozier O. (2003). Cytoscape: a software environment for integrated models of biomolecular interaction networks. *Genome Research*.

[56] del Sol A., Fujihashi H., Amoros D., Nussinov R. (2006). Residues crucial for maintaining short paths in network communication mediate signaling in proteins. *Molecular Systems Biology*.

[57] Brysbaert G., Lorgouilloux K., Vranken W. F., Lensink M. F. (2018). RINspector: a Cytoscape app for centrality analyses and DynaMine flexibility prediction. *Bioinformatics*.

[58] Shen Y., Maupetit J., Derreumaux P., Tufféry P. (2014). Improved PEP-FOLD approach for peptide and miniprotein structure prediction. *Journal of Chemical Theory and Computation*.

[59] Eswar N., Eramian D., Webb B., Shen M. Y., Sali A., Kobe B., Guss M., Huber T. (2008). Protein Structure Modeling with MODELLER. *Structural Proteomics. Methods in Molecular Biology™*.

[60] Chen V. B., Arendall W. B., Headd J. J. (2010). MolProbity: all-atom structure validation for macromolecular crystallography. *Acta Crystallographica Section D: Biological Crystallography*.

[61] Rodrigues C. H., Pires D. E., Ascher D. B. (2018). DynaMut: predicting the impact of mutations on protein conformation, flexibility and stability. *Nucleic Acids Research*.

[62] Montelione G. T., Nilges M., Bax A. (2013). Recommendations of the wwPDB NMR Validation Task Force. *Structure*.

[63] Tejero R., Snyder D., Mao B., Aramini J. M., Montelione G. T. (2013). PDBStat: a universal restraint converter and restraint analysis software package for protein NMR. *Journal of Biomolecular NMR*.

[64] Dahms S. O., Hardes K., Becker G. L., Steinmetzer T., Brandstetter H., Than M. E. (2014). X-ray structures of human furin in complex with competitive inhibitors. *ACS Chemical Biology*.

[65] Vankadari N. (2020). Structure of furin protease binding to SARS-CoV-2 spike glycoprotein and implications for potential targets and virulence. *The Journal of Physical Chemistry Letters*.

[66] Langnau C., Rohlfing A. K., Gekeler S. (2021). Platelet activation and plasma levels of furin are associated with prognosis of patients with coronary artery disease and COVID-19. *Arteriosclerosis, Thrombosis, and Vascular Biology*.

[67] Morrison C. (2018). Constrained peptides' time to shine?. *Nature Reviews Drug Discovery*.

[68] Falanga A., Nigro E., De Biasi M. G. (2017). Cyclic peptides as novel therapeutic microbicides: engineering of human defensin mimetics. *Molecules*.

[69] Ruchala P., Cho S., Cole A. L. (2011). Simplified *θ*-defensins: search for new antivirals. *International Journal of Peptide Research and Therapeutics*.

[70] Owen S. M., Rudolph D. L., Wang W. (2004). RC-101, a retrocyclin-1 analogue with enhanced activity against primary HIV type 1 isolates. *AIDS Research and Human Retroviruses*.

[71] Oh Y. T., Tran D., Buchanan T. A., Selsted M. E., Youn J. H. (2015). *θ*-Defensin RTD-1 improves insulin action and normalizes plasma glucose and FFA levels in diet-induced obese rats. *American Journal of Physiology-Endocrinology and Metabolism*.

[72] Conibear A. C., Bochen A., Rosengren K. J. (2014). The cyclic cystine ladder of theta-defensins as a stable, bifunctional scaffold: a proof-of-concept study using the integrin-binding RGD motif. *Chembiochem*.

[73] Panteleev P., Bolosov I., Balandin S., Ovchinnikova T. (2015). Structure and biological functions of *β*-hairpin antimicrobial peptides. *Acta Naturae (англоязычная версия)*.

[74] Selsted M. E., Ouellette A. J. (2005). Mammalian defensins in the antimicrobial immune response. *Nature Immunology*.

[75] Olli S., Nagaraj R., Motukupally S. R. (2015). A hybrid cationic peptide composed of human *β*-defensin-1 and humanized *Θ*-defensin sequences exhibits salt-resistant antimicrobial activity. *Antimicrobial Agents and Chemotherapy*.

[76] Wang W., Cole A. M., Hong T., Waring A. J., Lehrer R. I. (2003). Retrocyclin, an antiretroviral *θ*-defensin, is a lectin. *The Journal of Immunology*.

[77] Venkataraman N., Cole A. L., Ruchala P. (2009). Reawakening retrocyclins: ancestral human defensins active against HIV-1. *PLoS Biology*.

[78] Chalichem N. S. S., Bethapudi B., Mundkinajeddu D. (2020). Aminoglycosides can be a better choice over macrolides in COVID-19 regimen: plausible mechanism for repurposing strategy. *Medical Hypotheses*.

[79] Vabret N., Britton G. J., Gruber C. (2020). Immunology of COVID-19: current state of the science. *Immunity*.

[80] Liu Y., Sawalha A. H., Lu Q. (2021). COVID-19 and autoimmune diseases. *Current Opinion in Rheumatology*.

[81] Jamilloux Y., Henry T., Belot A. (2020). Should we stimulate or suppress immune responses in COVID-19? Cytokine and anti-cytokine interventions. *Autoimmunity Reviews*.

[82] Nadimi Parashkouhi S., Mosaddeghi P., Bagheri A. (2021). The dual sides of interferon induction in COVID-19 treatment. *Trends in Pharmaceutical Sciences*.

[83] Mosaddeghi P., Shahabinezhad F., Dehghani Z. (2021). Therapeutic approaches for COVID-19 based on the interferon-mediated immune responses. *Current Signal Transduction Therapy*.

[84] Tay M. Z., Poh C. M., Rénia L., MacAry P. A., Ng L. F. (2020). The trinity of COVID-19: immunity, inflammation and intervention. *Nature Reviews Immunology*.

[85] Schaal J. B., Maretzky T., Tran D. Q. (2018). Macrocyclic *θ*-defensins suppress tumor necrosis factor-*α* (TNF-*α*) shedding by inhibition of TNF-*α*-converting enzyme. *Journal of Biological Chemistry*.

[86] Tongaonkar P., Punj V., Subramanian A. (2019). RTD-1 therapeutically normalizes synovial gene signatures in rat autoimmune arthritis and suppresses proinflammatory mediators in RA synovial fibroblasts. *Physiological Genomics*.

[87] Tongaonkar P., Trinh K. K., Schaal J. B. (2015). Rhesus macaque *θ*-defensin RTD-1 inhibits proinflammatory cytokine secretion and gene expression by inhibiting the activation of NF-*κ*B and MAPK pathways. *Journal of Leukocyte Biology*.

[88] Bagheri A., Moezzi S. M. I., Mosaddeghi P. (2021). Interferon-inducer antivirals: potential candidates to combat COVID-19. *International Immunopharmacology*.

[89] Mahendran A. S. K., Lim Y. S., Fang C.-M., Loh H.-S., Le C. F. (2020). The potential of antiviral peptides as COVID-19 therapeutics. *Frontiers in Pharmacology*.

[90] Maiti B. K. (2020). Potential role of peptide-based antiviral therapy against SARS-CoV-2 infection. *ACS Pharmacology & Translational Science*.

[91] Ashaolu T. J., Nawaz A., Walayat N., Khalifa I. (2021). Potential “biopeptidal” therapeutics for severe respiratory syndrome coronaviruses: a review of antiviral peptides, viral mechanisms, and prospective needs. *Applied Microbiology and Biotechnology*.

[92] Zhao H., To K. K. W., Sze K. H. (2020). A broad-spectrum virus- and host-targeting peptide against respiratory viruses including influenza virus and SARS-CoV-2. *Nature Communications*.

[93] Wang C., Wang S., Li D. (2020). Lectin-like intestinal defensin inhibits 2019-nCoV spike binding to ACE2.

[94] Wang C., Wang S., Li D., Wei D.-Q., Zhao J., Wang J. (2020). Human intestinal defensin 5 inhibits SARS-CoV-2 invasion by cloaking ACE2. *Gastroenterology*.

[95] Kerget B., Kerget F., Aksakal A., Aşkın S., Sağlam L., Akgün M. (2021). Evaluation of alpha defensin, IL-1 receptor antagonist, and IL-18 levels in COVID-19 patients with macrophage activation syndrome and acute respiratory distress syndrome. *Journal of Medical Virology*.

[96] Zhao H. Z. J., Zhang K., Chu H. (2016). A novel peptide with potent and broad-spectrum antiviral activities against multiple respiratory viruses. *Scientific Reports*.

[97] https://clinicaltrials.gov/ct2/show/NCT04784897.

[98] https://clinicaltrials.gov/ct2/show/results/NCT04708236.

[99] Bakovic A., Risner K., Bhalla N. (2020). Brilacidin, a COVID-19 drug candidate, exhibits potent *in vitro* antiviral activity against SARS-CoV-2.

[100] Dolgin E. (2021). The race for antiviral drugs to beat COVID — and the next pandemic. *Nature*.

[101] Xu D., Lu W. (2020). Defensins: a double-edged sword in host immunity. *Frontiers in Immunology*.

[102] Abu-Fanne R., Stepanova V., Litvinov R. I. (2019). Neutrophil *α*-defensins promote thrombosis in vivo by altering fibrin formation, structure, and stability. *Blood*.

[103] Fureby A., Elofsson U., Gerde P. (2015). Pulmonary delivery of antimicrobial peptides. *ONdrugDelivery Magazine*.

