# Supplementary material for: Theta-Defensins to Counter COVID-19 as Furin Inhibitors: In Silico Efficiency Prediction and Novel Compound Design
Source: Comput Math Methods Med. 2022 Feb 9;2022:9735626. doi: 10.1155/2022/9735626 (PMC8829439; doi:10.1155/2022/9735626)
Supplement: Supplementary Materials — Supplementary Data 1. Figure S1: cartoon representation of furin cavities. Figure S2: the convexity index for each residue of theta defensins. Figure S3: defensin positive electrostatic potential. Figure S4: a centrality analysis of the important residues involved in the peptide-furin complex. Supplementary Data 2. Table S1: the available UniProtKB data on the furin reference sequence. Table S2: the interactive residues in furin-peptide complexes (available in PepDB). Table S3: the convexity index for each residue of theta defensins. The most protruded side chain is arginine. Table S4: furin-peptide docking, the structure-based method, resulted in ten different orientations for each peptide structure and furin structure. Table S5: designing a novel peptide, which is referred to as “construct.” The criteria for designing the construct are achieving a peptide with higher stability, lower antigenicity, and higher electrostatic potential. [file 9735626.f1.zip › Supplemetary Data - 2.docx]

| **Feature key** | **Position(s)** | **DescriptionActions** |
| --- | --- | --- |
| Metal bindingi | [115](https://www.uniprot.org/blast/?about=P09958%5b115%5d&key=Metal%20binding) | Calcium 1Combined sources2 Publications |
| Active sitei | [D153](https://www.uniprot.org/blast/?about=P09958%5b153%5d&key=Active%20site) | Charge relay systemPROSITE-ProRule annotation |
| Binding sitei | [154](https://www.uniprot.org/blast/?about=P09958%5b154%5d&key=Binding%20site) | SubstrateCombined sources2 Publications |
| Metal bindingi | [162](https://www.uniprot.org/blast/?about=P09958%5b162%5d&key=Metal%20binding) | Calcium 1Combined sources2 Publications |
| Metal bindingi | [174](https://www.uniprot.org/blast/?about=P09958%5b174%5d&key=Metal%20binding) | Calcium 2Combined sources2 Publications |
| Metal bindingi | [179](https://www.uniprot.org/blast/?about=P09958%5b179%5d&key=Metal%20binding) | Calcium 2Combined sources2 Publications |
| Metal bindingi | [181](https://www.uniprot.org/blast/?about=P09958%5b181%5d&key=Metal%20binding) | Calcium 2; via carbonyl oxygenCombined sources2 Publications |
| Active sitei | [194](https://www.uniprot.org/blast/?about=P09958%5b194%5d&key=Active%20site) | Charge relay systemPROSITE-ProRule annotation |
| Metal bindingi | [205](https://www.uniprot.org/blast/?about=P09958%5b205%5d&key=Metal%20binding) | Calcium 1; via carbonyl oxygenCombined sources2 Publications |
| Metal bindingi | [208](https://www.uniprot.org/blast/?about=P09958%5b208%5d&key=Metal%20binding) | Calcium 1Combined sources2 Publications |
| Metal bindingi | [210](https://www.uniprot.org/blast/?about=P09958%5b210%5d&key=Metal%20binding) | Calcium 1; via carbonyl oxygenCombined sources2 Publications |
| Metal bindingi | [212](https://www.uniprot.org/blast/?about=P09958%5b212%5d&key=Metal%20binding) | Calcium 1; via carbonyl oxygenCombined sources2 Publications |
| Binding sitei | [236](https://www.uniprot.org/blast/?about=P09958%5b236%5d&key=Binding%20site) | SubstrateCombined sources2 Publications |
| Metal bindingi | [258](https://www.uniprot.org/blast/?about=P09958%5b258%5d&key=Metal%20binding) | Calcium 3Combined sources2 Publications |
| Binding sitei | [264](https://www.uniprot.org/blast/?about=P09958%5b264%5d&key=Binding%20site) | SubstrateCombined sources2 Publications |
| Metal bindingi | [301](https://www.uniprot.org/blast/?about=P09958%5b301%5d&key=Metal%20binding) | Calcium 3Combined sources2 Publications |
| Binding sitei | [306](https://www.uniprot.org/blast/?about=P09958%5b306%5d&key=Binding%20site) | SubstrateCombined sources2 Publications |
| Binding sitei | [308](https://www.uniprot.org/blast/?about=P09958%5b308%5d&key=Binding%20site) | SubstrateCombined sources2 Publications |
| Metal bindingi | [331](https://www.uniprot.org/blast/?about=P09958%5b331%5d&key=Metal%20binding) | Calcium 3Combined sources2 Publications |
| Active sitei | [368](https://www.uniprot.org/blast/?about=P09958%5b368%5d&key=Active%20site) | Charge relay systemPROSITE-ProRule annotation |
| Binding sitei | [368](https://www.uniprot.org/blast/?about=P09958%5b368%5d&key=Binding%20site) | SubstrateCombined sources2 Publications |

*Table S1 The available UniProtKB data on the furin reference sequence*

*Table S2 the interactive residues in furin-peptide complexes (available in PepDB), approximately 30 residues of furin are involved in the interaction of the enzyme with short peptide. The interactive peptides are all including arginine with a length of 5-7 residues*

| 1HVZ |  |  |  |
| --- | --- | --- | --- |
| **Column1** | **Column2** | **Column3** | **Column4** |
| 1 | A | GLY | 3/64 |
| 2 | A | PHE | 8/98 |
| 3 | A | CYS | 2/48 |
| 4 | A | ARG | 22/549999 |
| 5 | A | CYS | 2/58 |
| 6 | A | LEU | 1/51 |
| 7 | A | CYS | 2/09 |
| 8 | A | ARG | 18/799999 |
| 9 | A | ARG | 31/35 |
| 10 | A | GLY | 3/36 |
| 11 | A | VAL | 8/810001 |
| 12 | A | CYS | 2/36 |
| 13 | A | ARG | 13/45 |
| 14 | A | CYS | 2/05 |
| 15 | A | ILE | 5/12 |
| 16 | A | CYS | 3/03 |
| 17 | A | THR | 4/04 |
| 18 | A | ARG | 44/130005 |
|  |  |  |  |
| 2LYF |  |  |  |
| **Column1** | **Column2** | **Column3** | **Column4** |
| 1 | A | GLY | 3/01 |
| 2 | A | PHE | 12/33 |
| 3 | A | CYS | 2/96 |
| 4 | A | ARG | 6/5 |
| 5 | A | CYS | 2/38 |
| 6 | A | LEU | 4/41 |
| 7 | A | CYS | 3/08 |
| 8 | A | ARG | 15/47 |
| 9 | A | ARG | 38/16 |
| 10 | A | GLY | 3/63 |
| 11 | A | VAL | 10/379999 |
| 12 | A | CYS | 3/66 |
| 13 | A | ARG | 10/099998 |
| 14 | A | CYS | 2/3 |
| 15 | A | ILE | 4/05 |
| 16 | A | CYS | 2/48 |
| 17 | A | THR | 5/32 |
| 18 | A | ARG | 28/68 |
|  |  |  |  |
| 2LZI |  |  |  |
| **Column1** | **Column2** | **Column3** | **Column4** |
| 1 | A | GLY | 5/01 |
| 2 | A | ILE | 14/190001 |
| 3 | A | CYS | 2/85 |
| 4 | A | ARG | 11/07 |
| 5 | A | CYS | 1/78 |
| 6 | A | ILE | 6/52 |
| 7 | A | CYS | 1/89 |
| 8 | A | GLY | 1/78 |
| 9 | A | ARG | 48/16 |
| 10 | A | ARG | 25/969997 |
| 11 | A | ILE | 12/11 |
| 12 | A | CYS | 1/61 |
| 13 | A | ARG | 14/56 |
| 14 | A | CYS | 1/84 |
| 15 | A | ILE | 6/08 |
| 16 | A | CYS | 3/71 |
| 17 | A | GLY | 2/31 |
| 18 | A | ARG | 63/930004 |
|  |  |  |  |
| 2M1P |  |  |  |
| **Column1** | **Column2** | **Column3** | **Column4** |
| 1 | A | GLY | 3/75 |
| 2 | A | VAL | 9/22 |
| 3 | A | CYS | 4/42 |
| 4 | A | ARG | 20/629999 |
| 6 | A | VAL | 4/3 |
| 7 | A | CYS | 3/94 |
| 8 | A | ARG | 16/279999 |
| 9 | A | ARG | 37/269997 |
| 10 | A | GLY | 3/57 |
| 11 | A | VAL | 9/849999 |
| 12 | A | CYS | 4/73 |
| 13 | A | ARG | 20/35 |
| 15 | A | VAL | 4/05 |
| 16 | A | CYS | 3/89 |
| 17 | A | ARG | 13/42 |
| 18 | A | ARG | 35/290005 |
|  |  |  |  |
| 2M2G |  |  |  |
| **Column1** | **Column2** | **Column3** | **Column4** |
| 1 | A | GLY | 4/31 |
| 2 | A | VAL | 11/96 |
| 4 | A | ARG | 18/93 |
| 5 | A | CYS | 3/04 |
| 6 | A | VAL | 2/48 |
| 7 | A | CYS | 3/45 |
| 8 | A | ARG | 22/52 |
| 9 | A | ARG | 40/269997 |
| 10 | A | GLY | 3/83 |
| 11 | A | VAL | 8/660001 |
| 12 | A | CYS | 3/78 |
| 13 | A | ARG | 9/54 |
| 14 | A | CYS | 3/72 |
| 15 | A | VAL | 4/599999 |
| 17 | A | ARG | 11/369999 |
| 18 | A | ARG | 46/790005 |
|  |  |  |  |
| 2M2H |  |  |  |
| **Column1** | **Column2** | **Column3** | **Column4** |
| 1 | A | GLY | 4/42 |
| 2 | A | VAL | 12/889998 |
| 4 | A | ARG | 13/209999 |
| 5 | A | CYS | 4/64 |
| 6 | A | VAL | 4/83 |
| 8 | A | ARG | 15/73 |
| 9 | A | ARG | 40/699997 |
| 10 | A | GLY | 4/34 |
| 11 | A | VAL | 12/53 |
| 13 | A | ARG | 15/910001 |
| 14 | A | CYS | 4/72 |
| 15 | A | VAL | 5/03 |
| 17 | A | ARG | 19/42 |
| 18 | A | ARG | 44/120003 |
|  |  |  |  |
| 2M2S |  |  |  |
| **Column1** | **Column2** | **Column3** | **Column4** |
| 1 | A | GLY | 3/74 |
| 2 | A | VAL | 7/91 |
| 3 | A | CYS | 4/41 |
| 4 | A | ARG | 18/679998 |
| 6 | A | VAL | 6/65 |
| 8 | A | ARG | 26/540005 |
| 9 | A | ARG | 45/630005 |
| 10 | A | GLY | 5/31 |
| 11 | A | VAL | 12/19 |
| 13 | A | ARG | 21/48 |
| 15 | A | VAL | 6/3 |
| 16 | A | CYS | 5/01 |
| 17 | A | ARG | 12/000001 |
| 18 | A | ARG | 37/449997 |
|  |  |  |  |
| 2M2X |  |  |  |
| **Column1** | **Column2** | **Column3** | **Column4** |
| 1 | A | GLY | 5/14 |
| 2 | A | VAL | 11/650001 |
| 4 | A | ARG | 21/74 |
| 6 | A | VAL | 8/7 |
| 8 | A | ARG | 31/289999 |
| 9 | A | ARG | 59/480003 |
| 10 | A | GLY | 5/35 |
| 11 | A | VAL | 14/500001 |
| 13 | A | ARG | 21/66 |
| 15 | A | VAL | 7/74 |
| 17 | A | ARG | 28/509998 |
| 18 | A | ARG | 47/059994 |
|  |  |  |  |
| 2M2Y |  |  |  |
| **Column1** | **Column2** | **Column3** | **Column4** |
| 1 | A | ARG | 10/469999 |
| 2 | A | CYS | 2/45 |
| 3 | A | VAL | 3/06 |
| 4 | A | CYS | 3/05 |
| 5 | A | ARG | 12/589999 |
| 6 | A | ARG | 44/129997 |
| 7 | A | GLY | 3/54 |
| 8 | A | VAL | 8/85 |
| 9 | A | CYS | 3/18 |
| 10 | A | ARG | 18/66 |
| 11 | A | CYS | 1/7 |
| 12 | A | VAL | 2/62 |
| 13 | A | CYS | 1/33 |
| 14 | A | ARG | 18/809999 |
| 15 | A | ARG | 43/32 |
| 16 | A | GLY | 3/32 |
| 17 | A | VAL | 11/73 |
| 18 | A | CYS | 3/54 |
|  |  |  |  |
| 2M77 |  |  |  |
| **Column1** | **Column2** | **Column3** | **Column4** |
| 1 | A | GLY | 3/74 |
| 2 | A | ASP | 7/43 |
| 3 | A | CYS | 3/49 |
| 4 | A | ARG | 16/75 |
| 5 | A | CYS | 2/35 |
| 6 | A | LEU | 3/41 |
| 7 | A | CYS | 2/81 |
| 8 | A | ARG | 13/490001 |
| 9 | A | ARG | 43/330002 |
| 10 | A | GLY | 3/47 |
| 11 | A | VAL | 10/17 |
| 12 | A | CYS | 3/3 |
| 13 | A | ARG | 9/869999 |
| 14 | A | CYS | 2/92 |
| 15 | A | ILE | 5/59 |
| 16 | A | CYS | 3/1 |
| 17 | A | THR | 7/07 |
| 18 | A | ARG | 57/180004 |
|  |  |  |  |
| 2M78 |  |  |  |
| **Column1** | **Column2** | **Column3** | **Column4** |
| 1 | A | GLY | 3/63 |
| 2 | A | PHE | 11/78 |
| 3 | A | CYS | 3/05 |
| 4 | A | ARG | 12/259999 |
| 5 | A | CYS | 2/34 |
| 6 | A | LEU | 2/82 |
| 7 | A | CYS | 3/43 |
| 8 | A | ARG | 13/23 |
| 9 | A | ARG | 47/600002 |
| 10 | A | GLY | 3/05 |
| 11 | A | ASP | 5/08 |
| 12 | A | CYS | 3/37 |
| 13 | A | ARG | 11/059999 |
| 14 | A | CYS | 2/26 |
| 15 | A | ILE | 5/43 |
| 16 | A | CYS | 3/07 |
| 17 | A | THR | 6/37 |
| 18 | A | ARG | 40/32 |
|  |  |  |  |
| 2M79 |  |  |  |
| **Column1** | **Column2** | **Column3** | **Column4** |
| 1 | A | GLY | 3/37 |
| 2 | A | ASP | 6/28 |
| 3 | A | CYS | 3/22 |
| 4 | A | ARG | 18/050001 |
| 5 | A | CYS | 2/89 |
| 6 | A | LEU | 5/64 |
| 7 | A | CYS | 3/02 |
| 8 | A | ARG | 23/940001 |
| 9 | A | ARG | 37/919998 |
| 10 | A | GLY | 3/36 |
| 11 | A | ASP | 6/52 |
| 12 | A | CYS | 4/08 |
| 13 | A | ARG | 19/370003 |
| 14 | A | CYS | 2/44 |
| 15 | A | ILE | 4/89 |
| 16 | A | CYS | 3/14 |
| 17 | A | THR | 6/409999 |
| 18 | A | ARG | 46/540005 |

*Table S 3 The convexity index for each residue of theta defensins. The most protruded side chain is Arginine.*

*Table S4 Furin-peptide docking The structure-based method resulted in ten different orientations for each peptide structure and furin structure. With a view to the docking scores, it can be observed that the minimal energies are not necessarily related to correct orientation*

| **Column1** | **Column2** | **Column3** | **Column4** |
| --- | --- | --- | --- |
| Poor rotamers | 0 | 0/00% | Goal: <0.3% |
| Favored rotamers | 15 | 88/24% | Goal: >98% |
| Ramachandran outliers | 0 | 0/00% | Goal: <0.05% |
| Ramachandran favored | 18 | 100/00% | Goal: >98% |
| Rama distribution Z-score | -1.39 ± 1.35 | Goal: abs(Z score) < 2 |  |
| Cβ deviations >0.25Å | 4 | 23/53% | Goal: 0 |
| Bad bonds: | 0 / 155 | 0/00% | Goal: 0% |
| Bad angles: | 6 / 202 | 2/97% | Goal: <0.1% |
| CA Geometry outliers | 0 | 0/00% | Goal: <0.5% |

*TableS5 designing a novel peptide; we refer to this as “construct”. The criteria for designing the construct are achieving a peptide with higher stability, lower antigenicity, and higher electrostatic potential. The obtained sequence pattern extracted from the library of theta defensins provided a scaffold for rational design. De novo method and homology modeling approach revealed slightly different structures, yet the homology modeling approach yielded a more satisfactory structure.*
