# Supplementary material for: Theta-Defensins to Counter COVID-19 as Furin Inhibitors: In Silico Efficiency Prediction and Novel Compound Design
Source: Comput Math Methods Med. 2022 Feb 9;2022:9735626. doi: 10.1155/2022/9735626 (PMC8829439; doi:10.1155/2022/9735626)
Supplement: Supplementary Materials — Supplementary Data 1. Figure S1: cartoon representation of furin cavities. Figure S2: the convexity index for each residue of theta defensins. Figure S3: defensin positive electrostatic potential. Figure S4: a centrality analysis of the important residues involved in the peptide-furin complex. Supplementary Data 2. Table S1: the available UniProtKB data on the furin reference sequence. Table S2: the interactive residues in furin-peptide complexes (available in PepDB). Table S3: the convexity index for each residue of theta defensins. The most protruded side chain is arginine. Table S4: furin-peptide docking, the structure-based method, resulted in ten different orientations for each peptide structure and furin structure. Table S5: designing a novel peptide, which is referred to as “construct.” The criteria for designing the construct are achieving a peptide with higher stability, lower antigenicity, and higher electrostatic potential. [file 9735626.f1.zip › Supplemetary Data - 3.docx]

| \| **Feature key** \| **Position(s)** \| **DescriptionActions** \| \| --- \| --- \| --- \| \| Metal bindingi \| [115](https://www.uniprot.org/blast/?about=P09958%5b115%5d&key=Metal%20binding) \| Calcium 1Combined sources2 Publications \| \| Active sitei \| [D153](https://www.uniprot.org/blast/?about=P09958%5b153%5d&key=Active%20site) \| Charge relay systemPROSITE-ProRule annotation \| \| Binding sitei \| [154](https://www.uniprot.org/blast/?about=P09958%5b154%5d&key=Binding%20site) \| SubstrateCombined sources2 Publications \| \| Metal bindingi \| [162](https://www.uniprot.org/blast/?about=P09958%5b162%5d&key=Metal%20binding) \| Calcium 1Combined sources2 Publications \| \| Metal bindingi \| [174](https://www.uniprot.org/blast/?about=P09958%5b174%5d&key=Metal%20binding) \| Calcium 2Combined sources2 Publications \| \| Metal bindingi \| [179](https://www.uniprot.org/blast/?about=P09958%5b179%5d&key=Metal%20binding) \| Calcium 2Combined sources2 Publications \| \| Metal bindingi \| [181](https://www.uniprot.org/blast/?about=P09958%5b181%5d&key=Metal%20binding) \| Calcium 2; via carbonyl oxygenCombined sources2 Publications \| \| Active sitei \| [194](https://www.uniprot.org/blast/?about=P09958%5b194%5d&key=Active%20site) \| Charge relay systemPROSITE-ProRule annotation \| \| Metal bindingi \| [205](https://www.uniprot.org/blast/?about=P09958%5b205%5d&key=Metal%20binding) \| Calcium 1; via carbonyl oxygenCombined sources2 Publications \| \| Metal bindingi \| [208](https://www.uniprot.org/blast/?about=P09958%5b208%5d&key=Metal%20binding) \| Calcium 1Combined sources2 Publications \| \| Metal bindingi \| [210](https://www.uniprot.org/blast/?about=P09958%5b210%5d&key=Metal%20binding) \| Calcium 1; via carbonyl oxygenCombined sources2 Publications \| \| Metal bindingi \| [212](https://www.uniprot.org/blast/?about=P09958%5b212%5d&key=Metal%20binding) \| Calcium 1; via carbonyl oxygenCombined sources2 Publications \| \| Binding sitei \| [236](https://www.uniprot.org/blast/?about=P09958%5b236%5d&key=Binding%20site) \| SubstrateCombined sources2 Publications \| \| Metal bindingi \| [258](https://www.uniprot.org/blast/?about=P09958%5b258%5d&key=Metal%20binding) \| Calcium 3Combined sources2 Publications \| \| Binding sitei \| [264](https://www.uniprot.org/blast/?about=P09958%5b264%5d&key=Binding%20site) \| SubstrateCombined sources2 Publications \| \| Metal bindingi \| [301](https://www.uniprot.org/blast/?about=P09958%5b301%5d&key=Metal%20binding) \| Calcium 3Combined sources2 Publications \| \| Binding sitei \| [306](https://www.uniprot.org/blast/?about=P09958%5b306%5d&key=Binding%20site) \| SubstrateCombined sources2 Publications \| \| Binding sitei \| [308](https://www.uniprot.org/blast/?about=P09958%5b308%5d&key=Binding%20site) \| SubstrateCombined sources2 Publications \| \| Metal bindingi \| [331](https://www.uniprot.org/blast/?about=P09958%5b331%5d&key=Metal%20binding) \| Calcium 3Combined sources2 Publications \| \| Active sitei \| [368](https://www.uniprot.org/blast/?about=P09958%5b368%5d&key=Active%20site) \| Charge relay systemPROSITE-ProRule annotation \| \| Binding sitei \| [368](https://www.uniprot.org/blast/?about=P09958%5b368%5d&key=Binding%20site) \| SubstrateCombined sources2 Publications \|   *Table S1 The available UniProtKB data on the furin reference sequence*  4OMC |
| --- | --- | --- | --- | --- | --- | --- | --- | --- | --- | --- | --- | --- | --- | --- | --- | --- | --- | --- | --- | --- | --- | --- | --- | --- | --- | --- | --- | --- | --- | --- | --- | --- | --- | --- | --- | --- | --- | --- | --- | --- | --- | --- | --- | --- | --- | --- | --- | --- | --- | --- | --- | --- | --- | --- | --- | --- | --- | --- | --- | --- | --- | --- | --- | --- | --- | --- |
| **Interacting peptide residues:** |
| H: 1, 2, 3, 4, 5, |
|  |
| **Interacting receptor residues:** |
| A: 153, 154, 191, 192, 194, 227, 228, 231, 232, 233, 236, 253, 254, 255, 256, 257, 258, 264, 265, 266, 267, 291, 292, 293, 294, 295, 306, 308, 309, 367, 368, |
|  |
| **Peptide sequence:** |
| >H |
| XRVRX |

| 4OMD |
| --- |
| **Interacting peptide residues:** |
| H: 1, 2, 3, 4, 5, |
|  |
| **Interacting receptor residues:** |
| A: 153, 154, 191, 192, 194, 227, 228, 231, 236, 253, 254, 255, 256, 257, 258, 264, 265, 266, 291, 292, 293, 294, 295, 306, 308, 309, 367, 368, |
|  |
| **Peptide sequence:** |
| >H |
| XRVRX |

|  |
| --- |
| 4RYD |
| **Interacting peptide residues:** |
| H: 1, 2, 3, 4, 5, |
|  |
| **Interacting receptor residues:** |
| A: 153, 154, 191, 192, 194, 227, 228, 231, 232, 233, 236, 253, 254, 255, 256, 257, 258, 264, 265, 266, 267, 291, 292, 293, 294, 295, 306, 308, 309, 367, 368, |
|  |
| **Peptide sequence:** |
| >H |
| XRXRX |

| 5JMO |
| --- |
| **Interacting peptide residues:** |
| G: 1, 2, 3, 4, 5, 6, |
|  |
| **Interacting receptor residues:** |
| A: 153, 154, 191, 192, 194, 227, 231, 236, 253, 254, 255, 256, 257, 258, 264, 265, 266, 291, 292, 293, 294, 295, 306, 308, 309, 365, 366, 367, 368, 369, |
|  |
| **Peptide sequence:** |
| >G |
| XRVKXX |

| 5JXH |
| --- |
| **Interacting peptide residues:** |
| H: 1, 2, 3, 4, 5, |
|  |
| **Interacting receptor residues:** |
| A: 153, 154, 191, 192, 194, 227, 228, 231, 232, 233, 236, 253, 254, 255, 256, 257, 258, 264, 265, 266, 267, 291, 292, 293, 294, 295, 306, 308, 309, 367, 368, |
|  |
| **Peptide sequence:** |
| >H |
| XRVRX |

|  |
| --- |
| 5JXJ |
| **Column1** |
| **Interacting peptide residues:** |
| H: 1, 2, 3, 4, 5, |
|  |
| **Interacting receptor residues:** |
| A: 153, 154, 191, 192, 194, 227, 228, 231, 232, 233, 236, 253, 254, 255, 256, 257, 258, 264, 265, 266, 267, 291, 292, 293, 294, 295, 306, 308, 309, 367, 368, |
|  |
| **Peptide sequence:** |
| >H |
| XRVRX |

| 6EQV |
| --- |
| **Interacting peptide residues:** |
| D: 1, 2, 3, 4, 5, |
|  |
| **Interacting receptor residues:** |
| A: 153, 154, 191, 192, 194, 227, 228, 231, 236, 253, 254, 255, 256, 257, 258, 264, 265, 291, 292, 293, 294, 295, 306, 308, 309, 367, 368, |
|  |
| **Peptide sequence:** |
| >D |
| XXVRX |

| 6EQW |
| --- |
| **Interacting peptide residues:** |
| D: 1, 2, 3, 4, 5, |
|  |
| **Interacting receptor residues:** |
| A: 153, 154, 191, 192, 194, 227, 228, 231, 236, 253, 254, 255, 256, 257, 258, 264, 265, 266, 291, 292, 293, 294, 295, 306, 308, 309, 367, 368, |
|  |
| **Peptide sequence:** |
| >D |
| XRXRX |

| 6EQX |
| --- |
| **Column1** |
| **Interacting peptide residues:** |
| D: 1, 2, 3, 4, 5, 6, |
|  |
| **Interacting receptor residues:** |
| A: 153, 154, 191, 192, 194, 227, 228, 229, 231, 232, 233, 236, 253, 254, 255, 256, 257, 258, 264, 265, 266, 267, 291, 292, 293, 294, 295, 306, 308, 309, 367, 368, |
|  |
| **Peptide sequence:** |
| >D |
| RRRVRX |

| 6HLB |
| --- |
| **Interacting peptide residues:** |
| B: 1, 2, 3, 4, 5, 6, 7, |
|  |
| **Interacting receptor residues:** |
| A: 153, 154, 191, 192, 194, 227, 228, 229, 231, 232, 233, 236, 253, 254, 255, 256, 257, 258, 264, 265, 266, 267, 291, 292, 293, 294, 295, 306, 308, 309, 367, 368, |
|  |
| **Peptide sequence:** |
| >B |
| RRRRXRX |

| 6HLD |
| --- |
| **Interacting peptide residues:** |
| B: 1, 2, 3, 4, 5, 6, 7, |
|  |
| **Interacting receptor residues:** |
| A: 153, 154, 191, 192, 194, 227, 229, 230, 231, 232, 233, 236, 253, 254, 255, 256, 257, 258, 264, 265, 266, 267, 291, 292, 293, 294, 295, 306, 308, 309, 367, 368, |
|  |
| **Peptide sequence:** |
| >B |
| XRRRXKX |

| 6HZA |
| --- |
| **Interacting peptide residues:** |
| B: 1, 2, 3, 4, 5, |
|  |
| **Interacting receptor residues:** |
| A: 153, 154, 191, 192, 194, 227, 228, 231, 232, 233, 236, 253, 254, 255, 256, 257, 258, 264, 265, 266, 267, 291, 292, 293, 294, 295, 306, 308, 309, 367, 368, |
|  |
| **Peptide sequence:** |
| >B |
| RRKRX |

| 6HZB |
| --- |
| **Column1** |
| **Interacting peptide residues:** |
| B: 1, 2, 3, 4, 5, |
|  |
| **Interacting receptor residues:** |
| A: 153, 154, 191, 192, 194, 227, 231, 232, 233, 236, 253, 254, 255, 256, 257, 258, 264, 265, 266, 267, 291, 292, 293, 294, 295, 306, 308, 309, 367, 368, |
|  |
| **Peptide sequence:** |
| >B |
| RRKKX |

| 6HZC |
| --- |
| **Interacting peptide residues:** |
| B: 1, 2, 3, 4, 5, 6, |
|  |
| **Interacting receptor residues:** |
| A: 153, 154, 191, 192, 194, 227, 231, 232, 233, 236, 253, 254, 255, 256, 257, 258, 264, 265, 266, 267, 291, 292, 293, 294, 295, 306, 308, 309, 367, 368, |
|  |
| **Peptide sequence:** |
| >B |
| KRRXKX |

| 6HZD |
| --- |
| **Interacting peptide residues:** |
| B: 1, 2, 3, 4, 5, 6, |
| Peptide sequence: |
| **Interacting receptor residues:** |
| A: 153, 154, 191, 192, 194, 227, 228, 229, 230, 231, 232, 233, 236, 253, 254, 255, 256, 257, 258, 264, 265, 266, 267, 291, 292, 293, 294, 295, 306, 308, 309, 367, 368, |
|  |
| **Peptide sequence:** |
| >B |
| RRRKRX |

*Table S2 the interactive residues in furin-peptide complexes (available in PepDB), approximately 30 residues of furin are involved in the interaction of the enzyme with short peptide. The interactive peptides are all including arginine with a length of 5-7 residues.*

| 1HVZ |  |  |  |
| --- | --- | --- | --- |
| **Column1** | **Column2** | **Column3** | **Column4** |
| 1 | A | GLY | 3/64 |
| 2 | A | PHE | 8/98 |
| 3 | A | CYS | 2/48 |
| 4 | A | ARG | 22/549999 |
| 5 | A | CYS | 2/58 |
| 6 | A | LEU | 1/51 |
| 7 | A | CYS | 2/09 |
| 8 | A | ARG | 18/799999 |
| 9 | A | ARG | 31/35 |
| 10 | A | GLY | 3/36 |
| 11 | A | VAL | 8/810001 |
| 12 | A | CYS | 2/36 |
| 13 | A | ARG | 13/45 |
| 14 | A | CYS | 2/05 |
| 15 | A | ILE | 5/12 |
| 16 | A | CYS | 3/03 |
| 17 | A | THR | 4/04 |
| 18 | A | ARG | 44/130005 |
|  |  |  |  |
| 2LYF |  |  |  |
| **Column1** | **Column2** | **Column3** | **Column4** |
| 1 | A | GLY | 3/01 |
| 2 | A | PHE | 12/33 |
| 3 | A | CYS | 2/96 |
| 4 | A | ARG | 6/5 |
| 5 | A | CYS | 2/38 |
| 6 | A | LEU | 4/41 |
| 7 | A | CYS | 3/08 |
| 8 | A | ARG | 15/47 |
| 9 | A | ARG | 38/16 |
| 10 | A | GLY | 3/63 |
| 11 | A | VAL | 10/379999 |
| 12 | A | CYS | 3/66 |
| 13 | A | ARG | 10/099998 |
| 14 | A | CYS | 2/3 |
| 15 | A | ILE | 4/05 |
| 16 | A | CYS | 2/48 |
| 17 | A | THR | 5/32 |
| 18 | A | ARG | 28/68 |
|  |  |  |  |
| 2LZI |  |  |  |
| **Column1** | **Column2** | **Column3** | **Column4** |
| 1 | A | GLY | 5/01 |
| 2 | A | ILE | 14/190001 |
| 3 | A | CYS | 2/85 |
| 4 | A | ARG | 11/07 |
| 5 | A | CYS | 1/78 |
| 6 | A | ILE | 6/52 |
| 7 | A | CYS | 1/89 |
| 8 | A | GLY | 1/78 |
| 9 | A | ARG | 48/16 |
| 10 | A | ARG | 25/969997 |
| 11 | A | ILE | 12/11 |
| 12 | A | CYS | 1/61 |
| 13 | A | ARG | 14/56 |
| 14 | A | CYS | 1/84 |
| 15 | A | ILE | 6/08 |
| 16 | A | CYS | 3/71 |
| 17 | A | GLY | 2/31 |
| 18 | A | ARG | 63/930004 |
|  |  |  |  |
| 2M1P |  |  |  |
| **Column1** | **Column2** | **Column3** | **Column4** |
| 1 | A | GLY | 3/75 |
| 2 | A | VAL | 9/22 |
| 3 | A | CYS | 4/42 |
| 4 | A | ARG | 20/629999 |
| 6 | A | VAL | 4/3 |
| 7 | A | CYS | 3/94 |
| 8 | A | ARG | 16/279999 |
| 9 | A | ARG | 37/269997 |
| 10 | A | GLY | 3/57 |
| 11 | A | VAL | 9/849999 |
| 12 | A | CYS | 4/73 |
| 13 | A | ARG | 20/35 |
| 15 | A | VAL | 4/05 |
| 16 | A | CYS | 3/89 |
| 17 | A | ARG | 13/42 |
| 18 | A | ARG | 35/290005 |
|  |  |  |  |
| 2M2G |  |  |  |
| **Column1** | **Column2** | **Column3** | **Column4** |
| 1 | A | GLY | 4/31 |
| 2 | A | VAL | 11/96 |
| 4 | A | ARG | 18/93 |
| 5 | A | CYS | 3/04 |
| 6 | A | VAL | 2/48 |
| 7 | A | CYS | 3/45 |
| 8 | A | ARG | 22/52 |
| 9 | A | ARG | 40/269997 |
| 10 | A | GLY | 3/83 |
| 11 | A | VAL | 8/660001 |
| 12 | A | CYS | 3/78 |
| 13 | A | ARG | 9/54 |
| 14 | A | CYS | 3/72 |
| 15 | A | VAL | 4/599999 |
| 17 | A | ARG | 11/369999 |
| 18 | A | ARG | 46/790005 |
|  |  |  |  |
| 2M2H |  |  |  |
| **Column1** | **Column2** | **Column3** | **Column4** |
| 1 | A | GLY | 4/42 |
| 2 | A | VAL | 12/889998 |
| 4 | A | ARG | 13/209999 |
| 5 | A | CYS | 4/64 |
| 6 | A | VAL | 4/83 |
| 8 | A | ARG | 15/73 |
| 9 | A | ARG | 40/699997 |
| 10 | A | GLY | 4/34 |
| 11 | A | VAL | 12/53 |
| 13 | A | ARG | 15/910001 |
| 14 | A | CYS | 4/72 |
| 15 | A | VAL | 5/03 |
| 17 | A | ARG | 19/42 |
| 18 | A | ARG | 44/120003 |
|  |  |  |  |
| 2M2S |  |  |  |
| **Column1** | **Column2** | **Column3** | **Column4** |
| 1 | A | GLY | 3/74 |
| 2 | A | VAL | 7/91 |
| 3 | A | CYS | 4/41 |
| 4 | A | ARG | 18/679998 |
| 6 | A | VAL | 6/65 |
| 8 | A | ARG | 26/540005 |
| 9 | A | ARG | 45/630005 |
| 10 | A | GLY | 5/31 |
| 11 | A | VAL | 12/19 |
| 13 | A | ARG | 21/48 |
| 15 | A | VAL | 6/3 |
| 16 | A | CYS | 5/01 |
| 17 | A | ARG | 12/000001 |
| 18 | A | ARG | 37/449997 |
|  |  |  |  |
| 2M2X |  |  |  |
| **Column1** | **Column2** | **Column3** | **Column4** |
| 1 | A | GLY | 5/14 |
| 2 | A | VAL | 11/650001 |
| 4 | A | ARG | 21/74 |
| 6 | A | VAL | 8/7 |
| 8 | A | ARG | 31/289999 |
| 9 | A | ARG | 59/480003 |
| 10 | A | GLY | 5/35 |
| 11 | A | VAL | 14/500001 |
| 13 | A | ARG | 21/66 |
| 15 | A | VAL | 7/74 |
| 17 | A | ARG | 28/509998 |
| 18 | A | ARG | 47/059994 |
|  |  |  |  |
| 2M2Y |  |  |  |
| **Column1** | **Column2** | **Column3** | **Column4** |
| 1 | A | ARG | 10/469999 |
| 2 | A | CYS | 2/45 |
| 3 | A | VAL | 3/06 |
| 4 | A | CYS | 3/05 |
| 5 | A | ARG | 12/589999 |
| 6 | A | ARG | 44/129997 |
| 7 | A | GLY | 3/54 |
| 8 | A | VAL | 8/85 |
| 9 | A | CYS | 3/18 |
| 10 | A | ARG | 18/66 |
| 11 | A | CYS | 1/7 |
| 12 | A | VAL | 2/62 |
| 13 | A | CYS | 1/33 |
| 14 | A | ARG | 18/809999 |
| 15 | A | ARG | 43/32 |
| 16 | A | GLY | 3/32 |
| 17 | A | VAL | 11/73 |
| 18 | A | CYS | 3/54 |
|  |  |  |  |
| 2M77 |  |  |  |
| **Column1** | **Column2** | **Column3** | **Column4** |
| 1 | A | GLY | 3/74 |
| 2 | A | ASP | 7/43 |
| 3 | A | CYS | 3/49 |
| 4 | A | ARG | 16/75 |
| 5 | A | CYS | 2/35 |
| 6 | A | LEU | 3/41 |
| 7 | A | CYS | 2/81 |
| 8 | A | ARG | 13/490001 |
| 9 | A | ARG | 43/330002 |
| 10 | A | GLY | 3/47 |
| 11 | A | VAL | 10/17 |
| 12 | A | CYS | 3/3 |
| 13 | A | ARG | 9/869999 |
| 14 | A | CYS | 2/92 |
| 15 | A | ILE | 5/59 |
| 16 | A | CYS | 3/1 |
| 17 | A | THR | 7/07 |
| 18 | A | ARG | 57/180004 |
|  |  |  |  |
| 2M78 |  |  |  |
| **Column1** | **Column2** | **Column3** | **Column4** |
| 1 | A | GLY | 3/63 |
| 2 | A | PHE | 11/78 |
| 3 | A | CYS | 3/05 |
| 4 | A | ARG | 12/259999 |
| 5 | A | CYS | 2/34 |
| 6 | A | LEU | 2/82 |
| 7 | A | CYS | 3/43 |
| 8 | A | ARG | 13/23 |
| 9 | A | ARG | 47/600002 |
| 10 | A | GLY | 3/05 |
| 11 | A | ASP | 5/08 |
| 12 | A | CYS | 3/37 |
| 13 | A | ARG | 11/059999 |
| 14 | A | CYS | 2/26 |
| 15 | A | ILE | 5/43 |
| 16 | A | CYS | 3/07 |
| 17 | A | THR | 6/37 |
| 18 | A | ARG | 40/32 |
|  |  |  |  |
| 2M79 |  |  |  |
| **Column1** | **Column2** | **Column3** | **Column4** |
| 1 | A | GLY | 3/37 |
| 2 | A | ASP | 6/28 |
| 3 | A | CYS | 3/22 |
| 4 | A | ARG | 18/050001 |
| 5 | A | CYS | 2/89 |
| 6 | A | LEU | 5/64 |
| 7 | A | CYS | 3/02 |
| 8 | A | ARG | 23/940001 |
| 9 | A | ARG | 37/919998 |
| 10 | A | GLY | 3/36 |
| 11 | A | ASP | 6/52 |
| 12 | A | CYS | 4/08 |
| 13 | A | ARG | 19/370003 |
| 14 | A | CYS | 2/44 |
| 15 | A | ILE | 4/89 |
| 16 | A | CYS | 3/14 |
| 17 | A | THR | 6/409999 |
| 18 | A | ARG | 46/540005 |

*Table S 3 The convexity index for each residue of theta defensins. The most protruded side chain is Arginine.*

| **Complex Template Information 1HVZ** | | | | | | | | | | |
| --- | --- | --- | --- | --- | --- | --- | --- | --- | --- | --- |
| **Column1** | **Column2** | **Column3** | **Column4** | **Column5** | **Column6** | **Column7** | **Column8** | **Column9** | **Column10** | **Column11** |
| **Molecule** | **PDB ID** | **Chain ID** | **Align_length** | **Coverage** | **Seq_ID (%)** | |  |  |  |  |
| Receptor | [4NE9](http://www.rcsb.org/pdb/explore/explore.do?structureId=4NE9) | B | 290 | 0.613 | 23.3 |  |  |  |  |  |
|  |  |  |  |  |  |  |  |  |  |  |
| **Summary of the Top 10 Models** |  |  |  |  |  |  |  |  |  |  |
| **Rank** | **1** | **2** | **3** | **4** | **5** | **6** | **7** | **8** | **9** | **10** |
| **Docking Score** | -221.83 | -219.81 | -211.33 | -207.46 | -206.22 | -204.4 | -204.2 | -204.1 | -203.55 | -200.98 |
| **Ligand rmsd (Å)** | 66.69 | 30.72 | 45.22 | 35.35 | 45.7 | 40.45 | 31.72 | 39.06 | 51.35 | 54.46 |
| (a) | Row 1: The ranks of the models. | | |  |  |  |  |  |  |  |
| (b) | Row 2: The docking energy scores. | | |  |  |  |  |  |  |  |
| (c) | Row 3: The ligand RMSDs from the input structures or modeled structures by homology modeling. | | | | | | | | |  |

| **Complex Template Information 2LYF** | | | | | | | | | | |
| --- | --- | --- | --- | --- | --- | --- | --- | --- | --- | --- |
| **Column1** | **Column2** | **Column3** | **Column4** | **Column5** | **Column6** | **Column7** | **Column8** | **Column9** | **Column10** | **Column11** |
| **Complex Template Information** |  |  |  |  |  |  |  |  |  |  |
| **Molecule** | **PDB ID** | **Chain ID** | **Align_length** | **Coverage** | **Seq_ID (%)** | |  |  |  |  |
| Receptor | [4NE9](http://www.rcsb.org/pdb/explore/explore.do?structureId=4NE9) | B | 290 | 0.613 | 23.3 |  |  |  |  |  |
|  |  |  |  |  |  |  |  |  |  |  |
| **Summary of the Top 10 Models** |  |  |  |  |  |  |  |  |  |  |
| **Rank** | **1** | **2** | **3** | **4** | **5** | **6** | **7** | **8** | **9** | **10** |
| **Docking Score** | -221.83 | -219.81 | -211.33 | -207.46 | -206.22 | -204.4 | -204.2 | -204.1 | -203.55 | -200.98 |
| **Ligand rmsd (Å)** | 66.69 | 30.72 | 45.22 | 35.35 | 45.7 | 40.45 | 31.72 | 39.06 | 51.35 | 54.46 |

| **2LZI** | | | | | | | | | | | |
| --- | --- | --- | --- | --- | --- | --- | --- | --- | --- | --- | --- |
| **Column1** | **Column2** | **Column3** | **Column4** | **Column5** | **Column6** | **Column7** | **Column8** | **Column9** | **Column10** | **Column11** |  |
| **Complex Template Information** |  |  |  |  |  |  |  |  |  |  |  |
| **Molecule** | **PDB ID** | **Chain ID** | **Align_length** | **Coverage** | **Seq_ID (%)** | |  |  |  |  |  |
| Receptor | [4OMC](http://www.rcsb.org/pdb/explore/explore.do?structureId=4OMC) | A | 473 | 1 | 100 |  |  |  |  |  |  |
|  |  |  |  |  |  |  |  |  |  |  |  |
| **Summary of the Top 10 Models** |  |  |  |  |  |  |  |  |  |  |  |
| **Rank** | **1** | **2** | **3** | **4** | **5** | **6** | **7** | **8** | **9** | **10** |  |
| **Docking Score** | -233.12 | -231.09 | -217.68 | -210.21 | -208.59 | -205.9 | -204.9 | -203.8 | -201.98 | -201.71 |  |
| **Ligand rmsd (Å)** | 72.51 | 73.78 | 67.16 | 73.08 | 66 | 68.32 | 70.99 | 67.02 | 39.05 | 65.26 |  |

| **2M1P** | | | | | | | | | | |
| --- | --- | --- | --- | --- | --- | --- | --- | --- | --- | --- |
| **Column1** | **Column2** | **Column3** | **Column4** | **Column5** | **Column6** | **Column7** | **Column8** | **Column9** | **Column10** | **Column11** |
| **Complex Template Information** |  |  |  |  |  |  |  |  |  |  |
| **Molecule** | **PDB ID** | **Chain ID** | **Align_length** | **Coverage** | **Seq_ID (%)** | |  |  |  |  |
| Receptor | [4OMC](http://www.rcsb.org/pdb/explore/explore.do?structureId=4OMC) | A | 473 | 1 | 100 |  |  |  |  |  |
|  |  |  |  |  |  |  |  |  |  |  |
| **Summary of the Top 10 Models** |  |  |  |  |  |  |  |  |  |  |
| **Rank** | **1** | **2** | **3** | **4** | **5** | **6** | **7** | **8** | **9** | **10** |
| **Docking Score** | -271.05 | -240.36 | -233.29 | -226.11 | -223.87 | -220.6 | -218.9 | -218 | -215.25 | -212.04 |
| **Ligand rmsd (Å)** | 65.6 | 66.08 | 72.85 | 70.21 | 64.61 | 65.2 | 71.63 | 56.58 | 64.51 | 55.63 |

| **2M2G** | | | | | | | | | | |
| --- | --- | --- | --- | --- | --- | --- | --- | --- | --- | --- |
| **Column1** | **Column2** | **Column3** | **Column4** | **Column5** | **Column6** | **Column7** | **Column8** | **Column9** | **Column10** | **Column11** |
| **Complex Template Information** |  |  |  |  |  |  |  |  |  |  |
| **Molecule** | **PDB ID** | **Chain ID** | **Align_length** | **Coverage** | **Seq_ID (%)** | |  |  |  |  |
| Receptor | [4OMC](http://www.rcsb.org/pdb/explore/explore.do?structureId=4OMC) | A | 473 | 1 | 100 |  |  |  |  |  |
|  |  |  |  |  |  |  |  |  |  |  |
| **Summary of the Top 10 Models** |  |  |  |  |  |  |  |  |  |  |
| **Rank** | **1** | **2** | **3** | **4** | **5** | **6** | **7** | **8** | **9** | **10** |
| **Docking Score** | -250.59 | -247.41 | -233.34 | -230.35 | -226 | -224.8 | -224.1 | -220.3 | -219.67 | -215.67 |
| **Ligand rmsd (Å)** | 66.62 | 67.38 | 72.36 | 65.38 | 70.67 | 67.03 | 43.01 | 63.72 | 79.97 | 27.13 |

| **2M2H** | | | | | | | | | | |
| --- | --- | --- | --- | --- | --- | --- | --- | --- | --- | --- |
| **Column1** | **Column2** | **Column3** | **Column4** | **Column5** | **Column6** | **Column7** | **Column8** | **Column9** | **Column10** | **Column11** |
| **Complex Template Information** |  |  |  |  |  |  |  |  |  |  |
| **Molecule** | **PDB ID** | **Chain ID** | **Align_length** | **Coverage** | **Seq_ID (%)** | |  |  |  |  |
| Receptor | [4OMC](http://www.rcsb.org/pdb/explore/explore.do?structureId=4OMC) | A | 473 | 1 | 100 |  |  |  |  |  |
|  |  |  |  |  |  |  |  |  |  |  |
| **Summary of the Top 10 Models** |  |  |  |  |  |  |  |  |  |  |
| **Rank** | **1** | **2** | **3** | **4** | **5** | **6** | **7** | **8** | **9** | **10** |
| **Docking Score** | -227.87 | -227.7 | -226.48 | -223.81 | -220.79 | -218.4 | -217.9 | -217.8 | -217.43 | -216.92 |
| **Ligand rmsd (Å)** | 65.67 | 69.71 | 68.54 | 73.03 | 72.32 | 65.22 | 77 | 65.21 | 72.73 | 67.14 |

| **2M2S** | | | | | | | | | | | |
| --- | --- | --- | --- | --- | --- | --- | --- | --- | --- | --- | --- |
| **Column1** | **Column2** | **Column3** | **Column4** | **Column5** | **Column6** | **Column7** | **Column8** | **Column9** | **Column10** | **Column11** |  |
| **Complex Template Information** |  |  |  |  |  |  |  |  |  |  |  |
| **Molecule** | **PDB ID** | **Chain ID** | **Align_length** | **Coverage** | **Seq_ID (%)** | |  |  |  |  |  |
| Receptor | [4OMC](http://www.rcsb.org/pdb/explore/explore.do?structureId=4OMC) | A | 473 | 1 | 100 |  |  |  |  |  |  |
|  |  |  |  |  |  |  |  |  |  |  |  |
| **Summary of the Top 10 Models** |  |  |  |  |  |  |  |  |  |  |  |
| **Rank** | **1** | **2** | **3** | **4** | **5** | **6** | **7** | **8** | **9** | **10** |  |
| **Docking Score** | -237.57 | -237.23 | -235.45 | -231.64 | -228.62 | -223.7 | -223.5 | -219 | -217.43 | -217.18 |  |
| **Ligand rmsd (Å)** | 66.69 | 77.31 | 65.7 | 69.13 | 71.97 | 65.69 | 70.64 | 73.56 | 65.55 | 79.78 |  |

| **2M2X** | | | | | | | | | | |
| --- | --- | --- | --- | --- | --- | --- | --- | --- | --- | --- |
| **Column1** | **Column2** | **Column3** | **Column4** | **Column5** | **Column6** | **Column7** | **Column8** | **Column9** | **Column10** | **Column11** |
| **Complex Template Information** |  |  |  |  |  |  |  |  |  |  |
| **Molecule** | **PDB ID** | **Chain ID** | **Align_length** | **Coverage** | **Seq_ID (%)** | |  |  |  |  |
| Receptor | [4OMC](http://www.rcsb.org/pdb/explore/explore.do?structureId=4OMC) | A | 473 | 1 | 100 |  |  |  |  |  |
|  |  |  |  |  |  |  |  |  |  |  |
| **Summary of the Top 10 Models** |  |  |  |  |  |  |  |  |  |  |
| **Rank** | **1** | **2** | **3** | **4** | **5** | **6** | **7** | **8** | **9** | **10** |
| **Docking Score** | -252.96 | -242.44 | -241.95 | -233.19 | -226.71 | -221.3 | -219.5 | -213.5 | -213.2 | -212.81 |
| **Ligand rmsd (Å)** | 69.37 | 65.78 | 64.95 | 66.42 | 71.55 | 73.95 | 74.22 | 64.18 | 65.74 | 64.65 |

| **2M2Y** | | | | | | | | | | | |
| --- | --- | --- | --- | --- | --- | --- | --- | --- | --- | --- | --- |
| **Column1** | **Column2** | **Column3** | **Column4** | **Column5** | **Column6** | **Column7** | **Column8** | **Column9** | **Column10** | **Column11** |  |
| **Complex Template Information** |  |  |  |  |  |  |  |  |  |  |  |
| **Molecule** | **PDB ID** | **Chain ID** | **Align_length** | **Coverage** | **Seq_ID (%)** | |  |  |  |  |  |
| Receptor | [4OMC](http://www.rcsb.org/pdb/explore/explore.do?structureId=4OMC) | A | 473 | 1 | 100 |  |  |  |  |  |  |
|  |  |  |  |  |  |  |  |  |  |  |  |
| **Summary of the Top 10 Models** |  |  |  |  |  |  |  |  |  |  |  |
| **Rank** | **1** | **2** | **3** | **4** | **5** | **6** | **7** | **8** | **9** | **10** |  |
| **Docking Score** | -248.55 | -237.81 | -224.66 | -223.06 | -221.46 | -221.3 | -219.6 | -219.5 | -214.04 | -212.34 |  |
| **Ligand rmsd (Å)** | 76.31 | 73.74 | 72.27 | 45.87 | 73.3 | 67.83 | 65.39 | 71.09 | 61.19 | 75.06 |  |

| **2M77** | | | | | | | | | | |
| --- | --- | --- | --- | --- | --- | --- | --- | --- | --- | --- |
| **Column1** | **Column2** | **Column3** | **Column4** | **Column5** | **Column6** | **Column7** | **Column8** | **Column9** | **Column10** | **Column11** |
| **Complex Template Information** |  |  |  |  |  |  |  |  |  |  |
| **Molecule** | **PDB ID** | **Chain ID** | **Align_length** | **Coverage** | **Seq_ID (%)** | |  |  |  |  |
| Receptor | [4NE9](http://www.rcsb.org/pdb/explore/explore.do?structureId=4NE9) | B | 290 | 0.613 | 23.3 |  |  |  |  |  |
|  |  |  |  |  |  |  |  |  |  |  |
| **Summary of the Top 10 Models** |  |  |  |  |  |  |  |  |  |  |
| **Rank** | **1** | **2** | **3** | **4** | **5** | **6** | **7** | **8** | **9** | **10** |
| **Docking Score** | -223.67 | -221.49 | -215.76 | -212.32 | -200.88 | -196.3 | -194.8 | -189.3 | -187.17 | -186.79 |
| **Ligand rmsd (Å)** | 31.47 | 60.6 | 53.52 | 25.4 | 55.57 | 55.18 | 35.45 | 53.14 | 30.96 | 39.15 |

| **2M88** | | | | | | | | | | |
| --- | --- | --- | --- | --- | --- | --- | --- | --- | --- | --- |
| **Column1** | **Column2** | **Column3** | **Column4** | **Column5** | **Column6** | **Column7** | **Column8** | **Column9** | **Column10** | **Column11** |
| **Complex Template Information** |  |  |  |  |  |  |  |  |  |  |
| **Molecule** | **PDB ID** | **Chain ID** | **Align_length** | **Coverage** | **Seq_ID (%)** | |  |  |  |  |
| Receptor | [2W2N](http://www.rcsb.org/pdb/explore/explore.do?structureId=2W2N) | A | 290 | 0.613 | 23.32W2Q |  |  |  |  |  |
| Ligand | [A](http://www.rcsb.org/pdb/explore/explore.do?structureId=A) | 290 | 0.613 | 23.32W2M | A |  |  |  |  |  |
|  |  |  |  |  |  |  |  |  |  |  |
| **Summary of the Top 10 Models** |  |  |  |  |  |  |  |  |  |  |
| **Rank** | **1** | **2** | **3** | **4** | **5** | **6** | **7** | **8** | **9** | **10** |
| **Docking Score** | -244.4 | -225.92 | -225.04 | -207.44 | -207.39 | -204.9 | -203.3 | -200.1 | -196.36 | -194.4 |
| **Ligand rmsd (Å)** | 46.37 | 48.83 | 59.1 | 73.86 | 51.21 | 53.97 | 59.14 | 48.65 | 70.79 | 50.04 |

| **2M79** | | | | | | | | | | |
| --- | --- | --- | --- | --- | --- | --- | --- | --- | --- | --- |
| **Column1** | **Column2** | **Column3** | **Column4** | **Column5** | **Column6** | **Column7** | **Column8** | **Column9** | **Column10** | **Column11** |
| **Complex Template Information** |  |  |  |  |  |  |  |  |  |  |
| **Molecule** | **PDB ID** | **Chain ID** | **Align_length** | **Coverage** | **Seq_ID (%)** | |  |  |  |  |
| Receptor | [2W2N](http://www.rcsb.org/pdb/explore/explore.do?structureId=2W2N) | A | 290 | 0.613 | 23.32W2Q |  |  |  |  |  |
| Ligand | [A](http://www.rcsb.org/pdb/explore/explore.do?structureId=A) | 290 | 0.613 | 23.32W2M | A |  |  |  |  |  |
|  |  |  |  |  |  |  |  |  |  |  |
| **Summary of the Top 10 Models** |  |  |  |  |  |  |  |  |  |  |
| **Rank** | **1** | **2** | **3** | **4** | **5** | **6** | **7** | **8** | **9** | **10** |
| **Docking Score** | -210.44 | -202.04 | -196.34 | -188.92 | -187.55 | -187 | -186.6 | -186.3 | -186.11 | -184.59 |
| **Ligand rmsd (Å)** | 51.44 | 41.21 | 40.6 | 41.25 | 52.82 | 44.36 | 50.81 | 27.88 | 49.17 | 48.3 |

Final constract

| **RGVCRCICRRGRGVCRCICRRG** |  |  |  |  |  |  |  |  |  |  |
| --- | --- | --- | --- | --- | --- | --- | --- | --- | --- | --- |
| **Column1** | **Column2** | **Column3** | **Column4** | **Column5** | **Column6** | **Column7** | **Column8** | **Column9** | **Column10** | **Column11** |
| **Complex Template Information** |  |  |  |  |  |  |  |  |  |  |
| **Molecule** | **PDB ID** | **Chain ID** | **Align_length** | **Coverage** | **Seq_ID (%)** | |  |  |  |  |
| Receptor | [4OMC](http://www.rcsb.org/pdb/explore/explore.do?structureId=4OMC) | A | 473 | 0.975 | 100 |  |  |  |  |  |
|  |  |  |  |  |  |  |  |  |  |  |
| **Summary of the Top 10 Models** |  |  |  |  |  |  |  |  |  |  |
| **Rank** | **1** | **2** | **3** | **4** | **5** | **6** | **7** | **8** | **9** | **10** |
| **Docking Score** | -263.2 | -230.51 | -230.16 | -230.06 | -224.93 | -221.9 | -215.8 | -215.5 | -214.39 | -214.27 |
| **Ligand rmsd (Å)** | 66.01 | 55.96 | 65.24 | 62.42 | 60.49 | 52.01 | 68.52 | 65.05 | 64.76 | 71.14 |

*Table S4 Furin-peptide docking The structure-based method resulted in ten different orientations for each peptide structure and furin structure. With a view to the docking scores, it can be observed that the minimal energies are not necessarily related to correct orientation*

| **Column1** | **Column2** | **Column3** | **Column4** |
| --- | --- | --- | --- |
| Poor rotamers | 0 | 0/00% | Goal: <0.3% |
| Favored rotamers | 15 | 88/24% | Goal: >98% |
| Ramachandran outliers | 0 | 0/00% | Goal: <0.05% |
| Ramachandran favored | 18 | 100/00% | Goal: >98% |
| Rama distribution Z-score | -1.39 ± 1.35 | Goal: abs(Z score) < 2 |  |
| Cβ deviations >0.25Å | 4 | 23/53% | Goal: 0 |
| Bad bonds: | 0 / 155 | 0/00% | Goal: 0% |
| Bad angles: | 6 / 202 | 2/97% | Goal: <0.1% |
| CA Geometry outliers | 0 | 0/00% | Goal: <0.5% |

*TableS5 designing a novel peptide; we refer to this as “construct”. The criteria for designing the construct are achieving a peptide with higher stability, lower antigenicity, and higher electrostatic potential. The obtained sequence pattern extracted from the library of theta defensins provided a scaffold for rational design. De novo method and homology modeling approach revealed slightly different structures, yet the homology modeling approach yielded a more satisfactory structure.*
