# Supplementary material for: Theta-Defensins to Counter COVID-19 as Furin Inhibitors: In Silico Efficiency Prediction and Novel Compound Design
Source: Comput Math Methods Med. 2022 Feb 9;2022:9735626. doi: 10.1155/2022/9735626 (PMC8829439; doi:10.1155/2022/9735626)
Supplement: Supplementary Materials — Supplementary Data 1. Figure S1: cartoon representation of furin cavities. Figure S2: the convexity index for each residue of theta defensins. Figure S3: defensin positive electrostatic potential. Figure S4: a centrality analysis of the important residues involved in the peptide-furin complex. Supplementary Data 2. Table S1: the available UniProtKB data on the furin reference sequence. Table S2: the interactive residues in furin-peptide complexes (available in PepDB). Table S3: the convexity index for each residue of theta defensins. The most protruded side chain is arginine. Table S4: furin-peptide docking, the structure-based method, resulted in ten different orientations for each peptide structure and furin structure. Table S5: designing a novel peptide, which is referred to as “construct.” The criteria for designing the construct are achieving a peptide with higher stability, lower antigenicity, and higher electrostatic potential. [file 9735626.f1.zip › Supplemetary Data 1.docx]

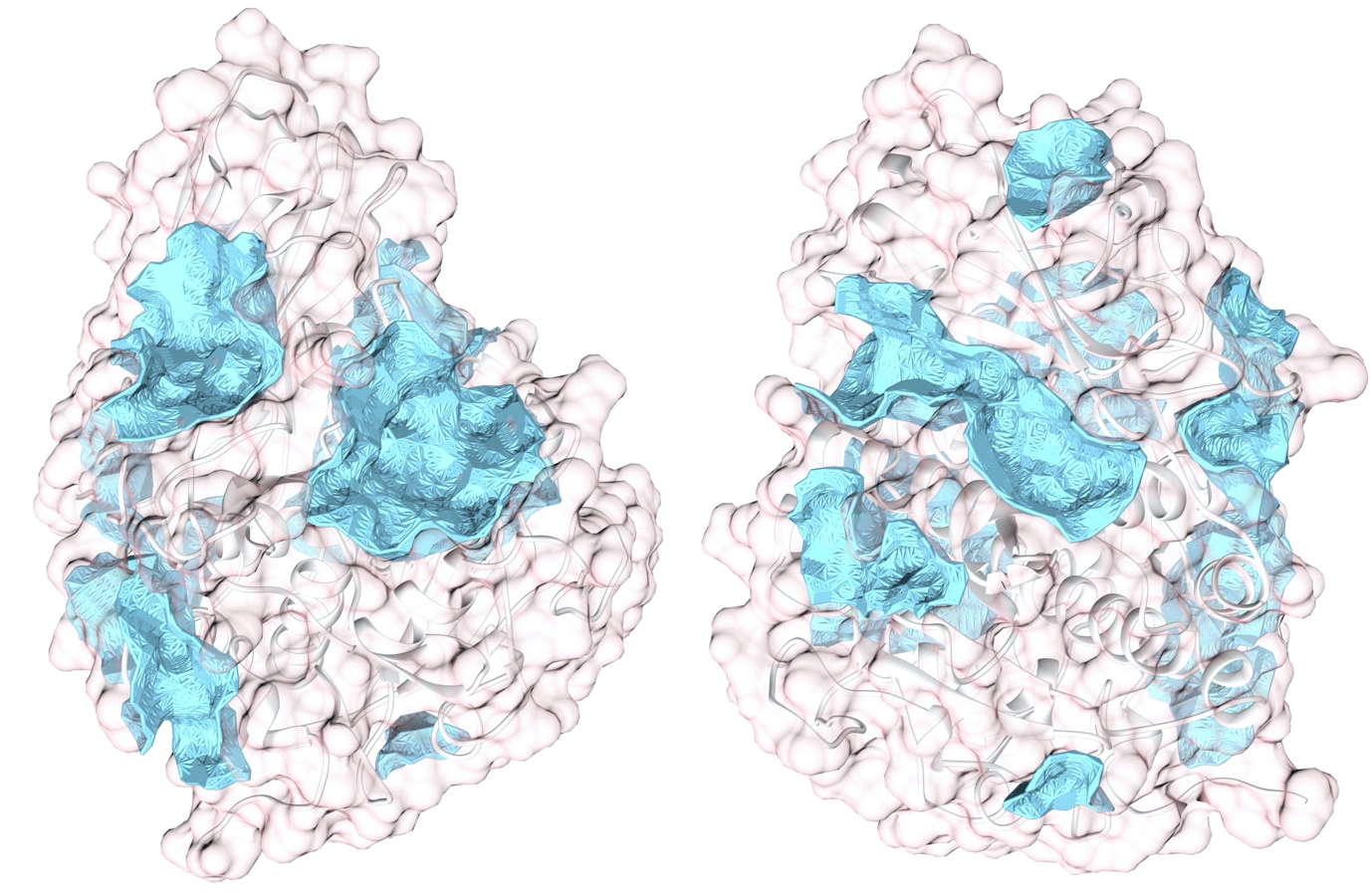


*Figure S 1 Cartoon representation of furin cavities At least 9 cavities were recognized on the surface of Furin (PDB entry: 5JXG)*


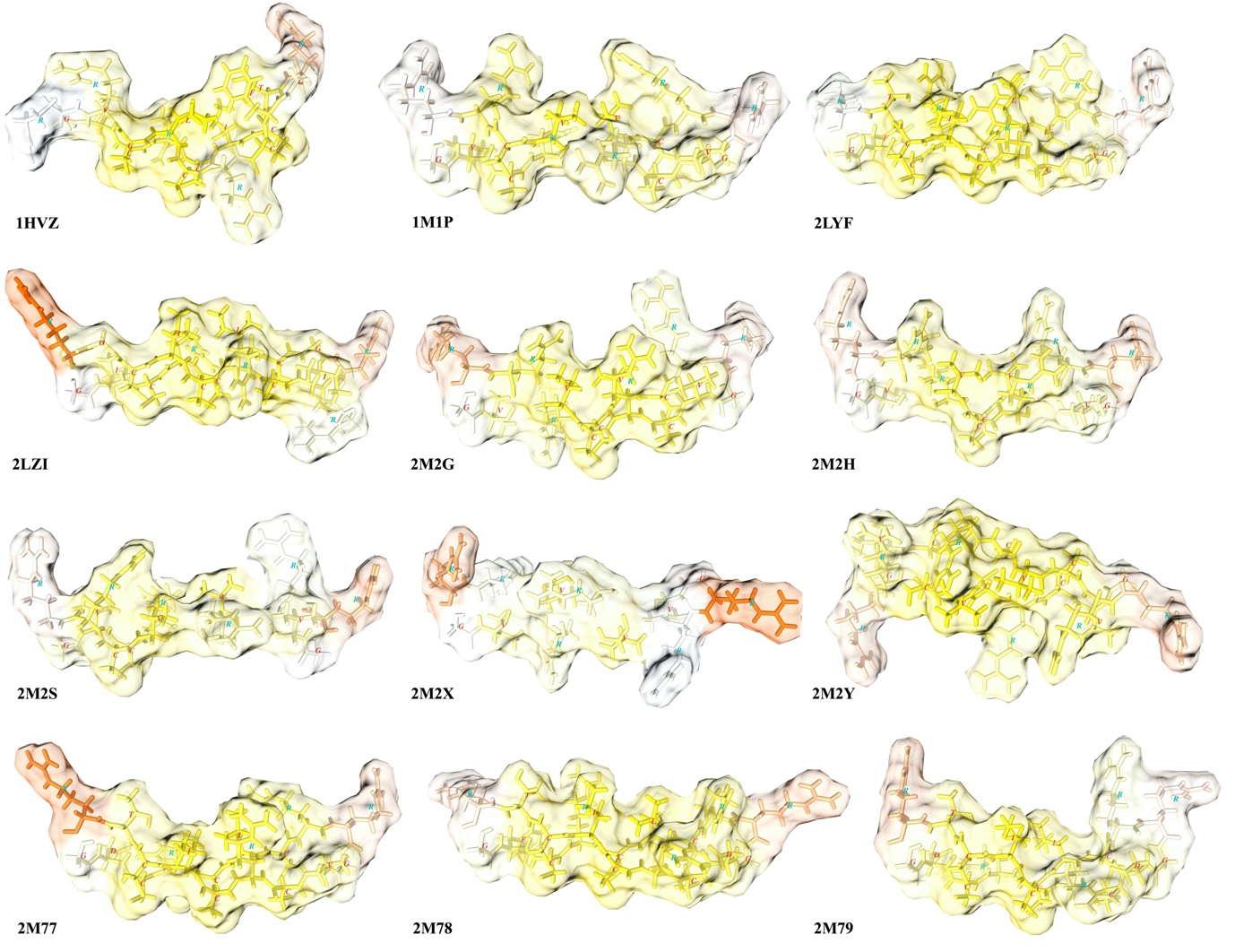


Figure S 2 The convexity index for each residue of theta defensins. The most protruded side chain is arginine.


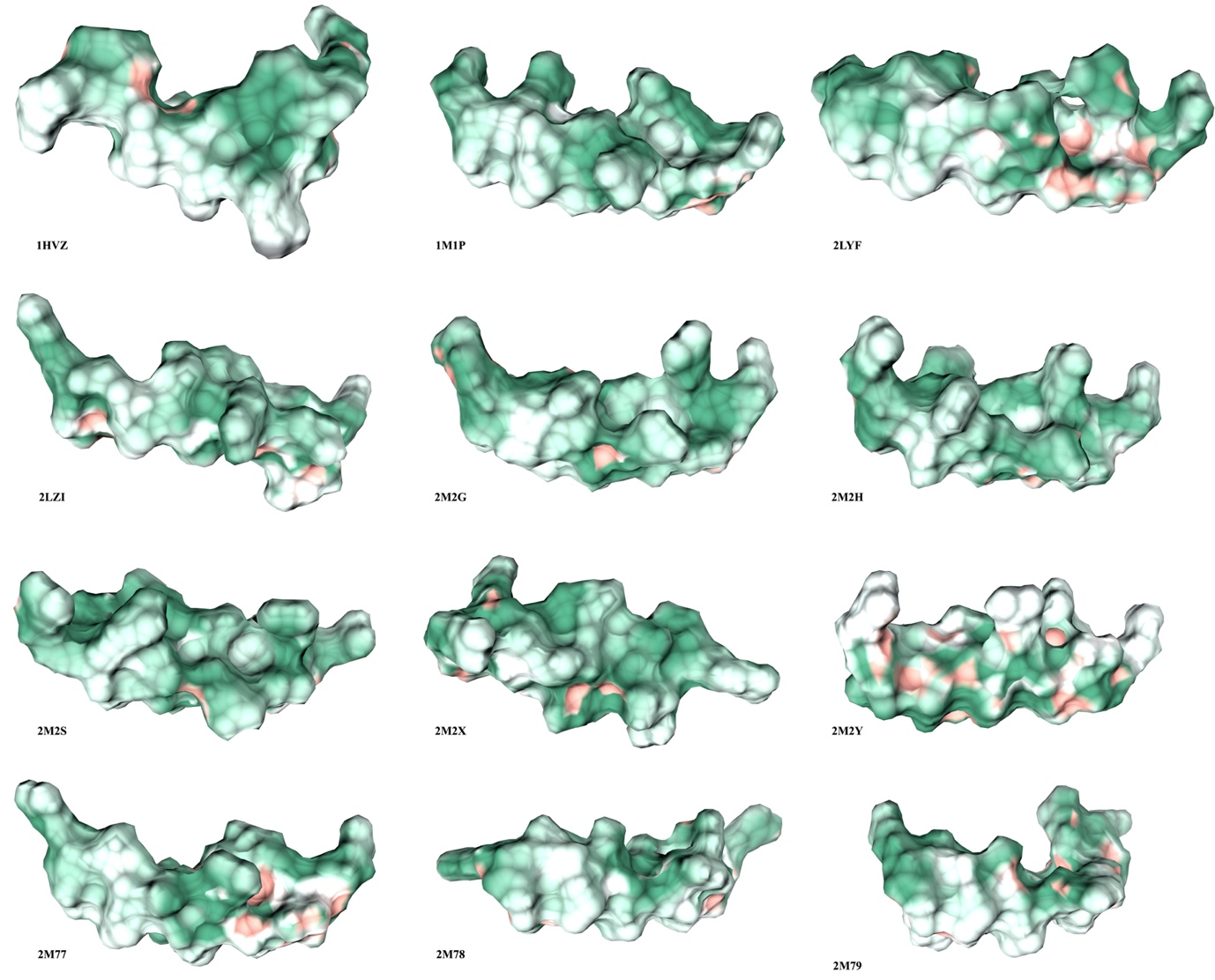


*Figure S 3 all defensins are highly positive in the term of electrostatic potential. A positive electrostatic complement peptide would engage the highly negative furin cleft.*


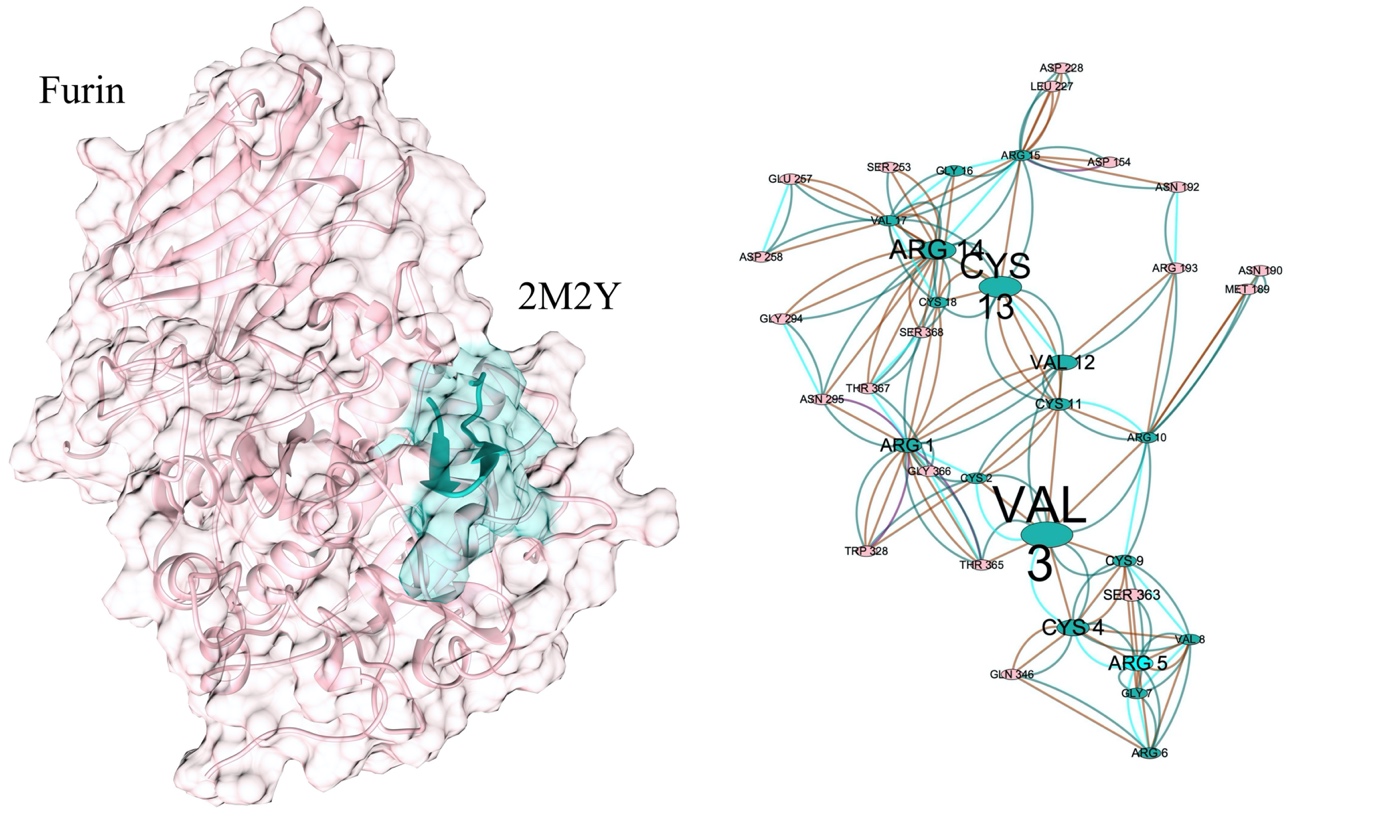

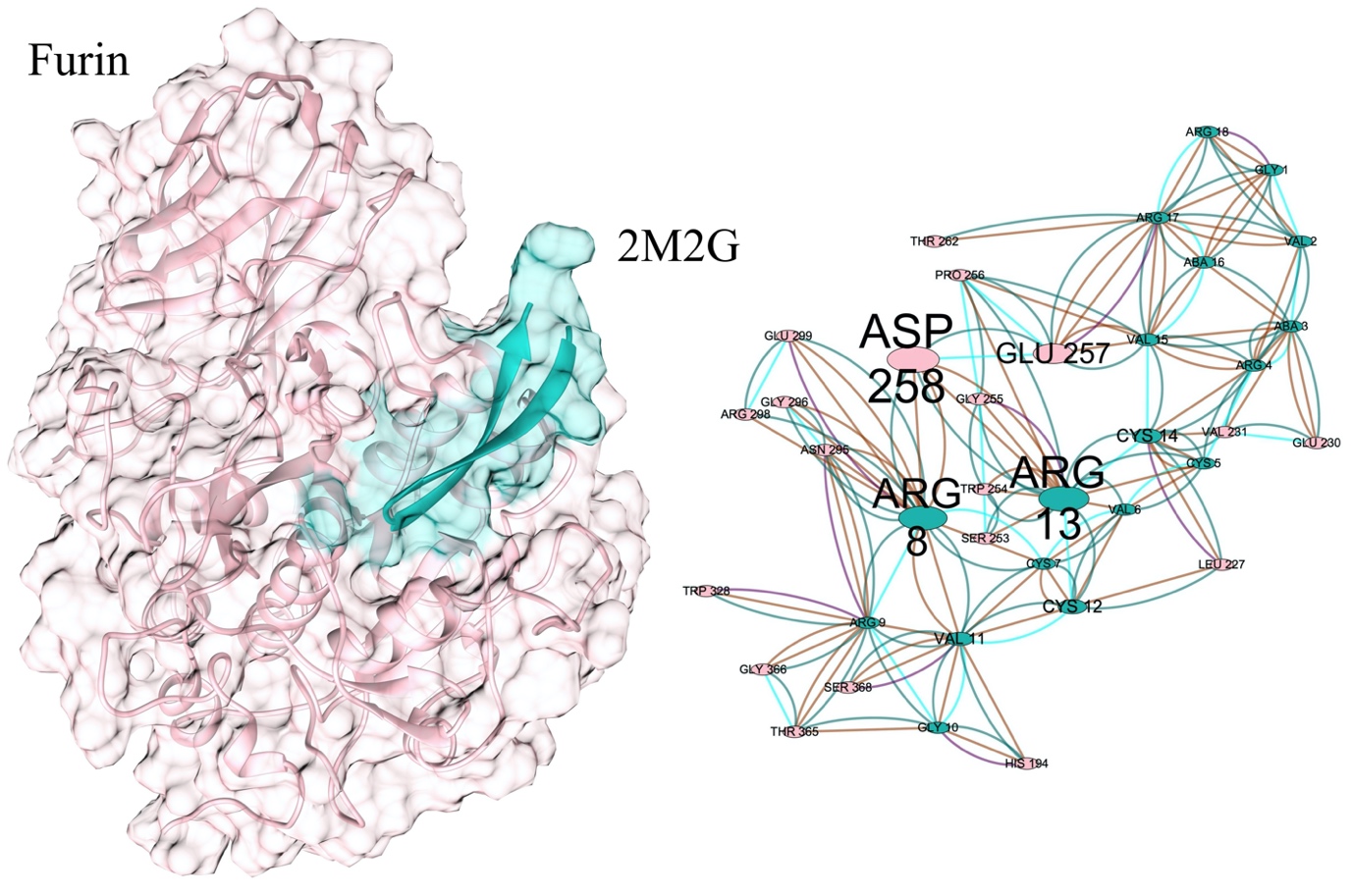

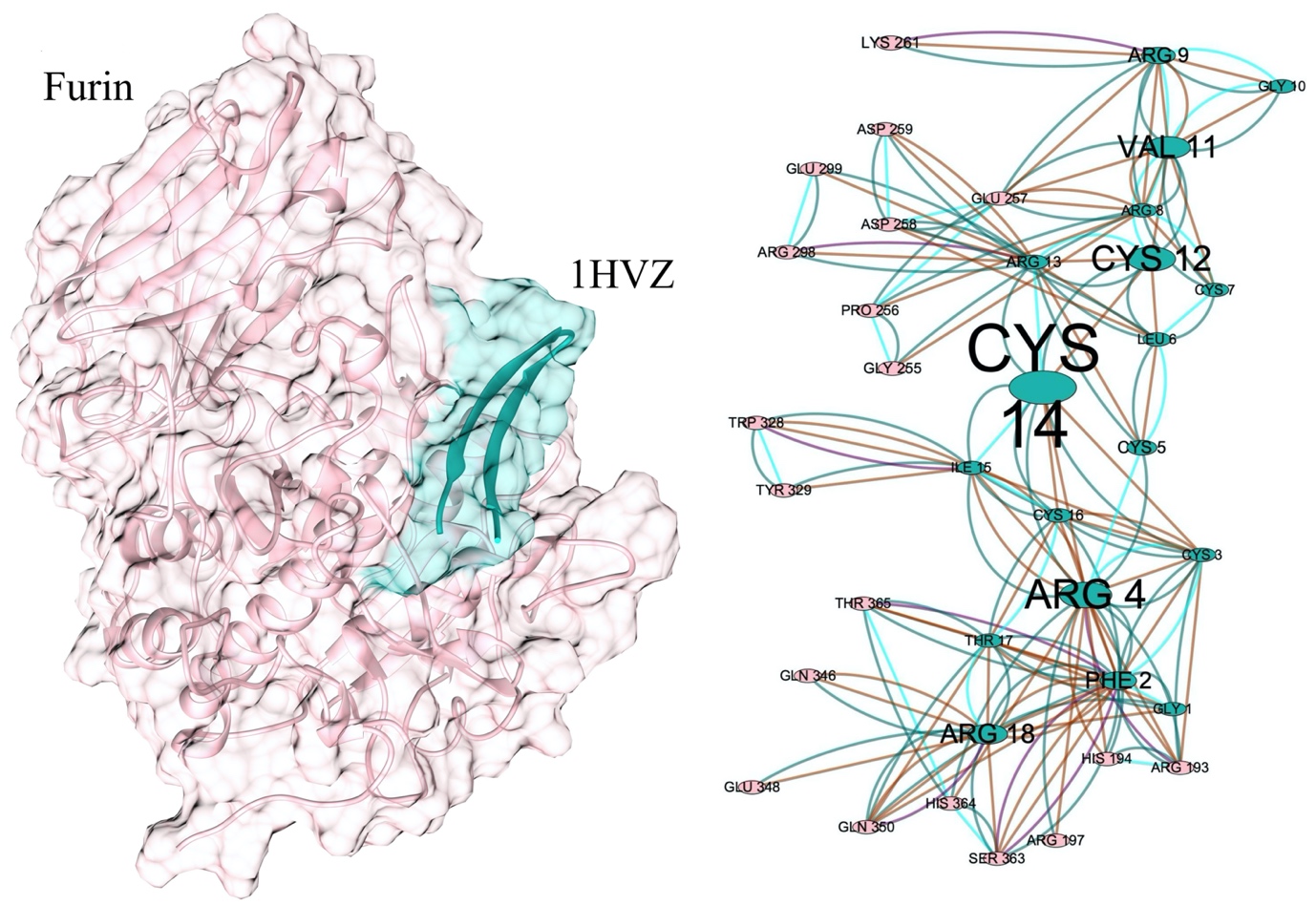

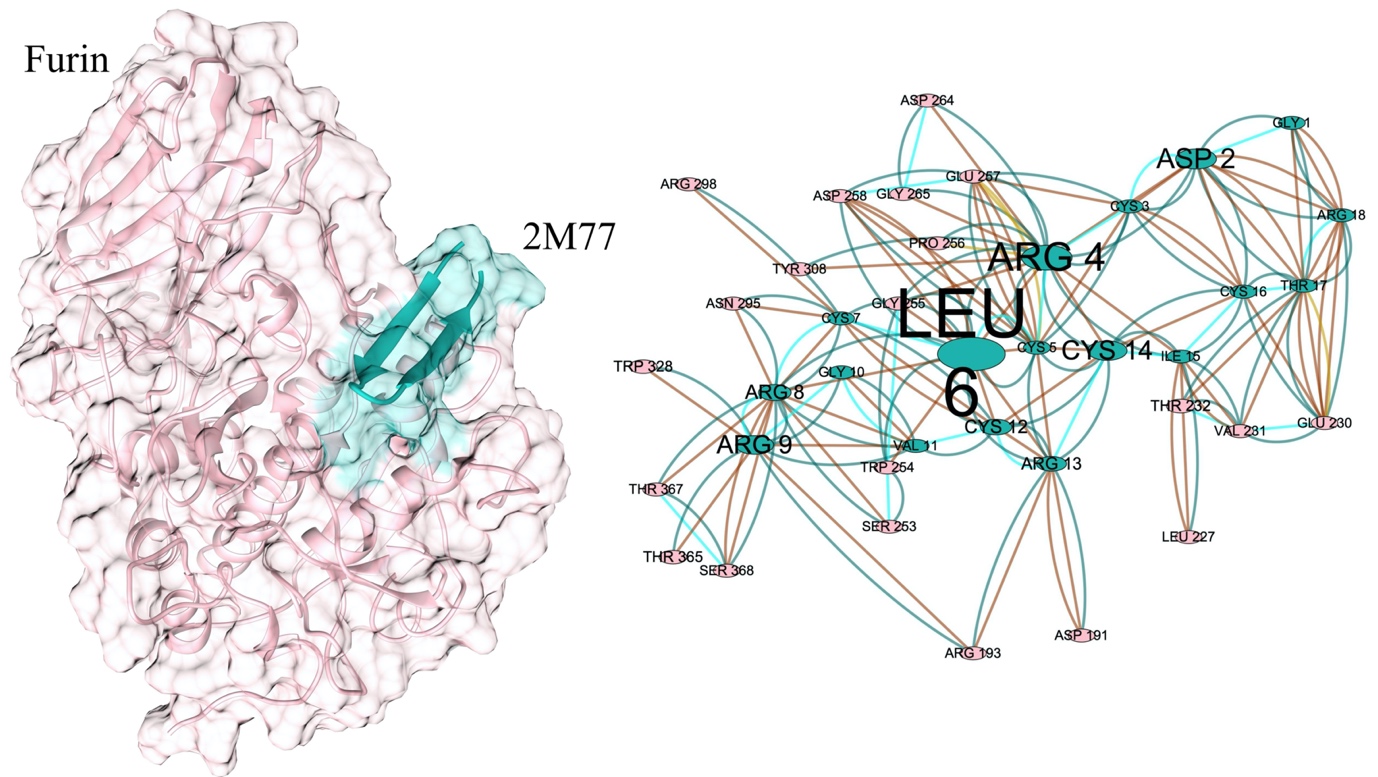

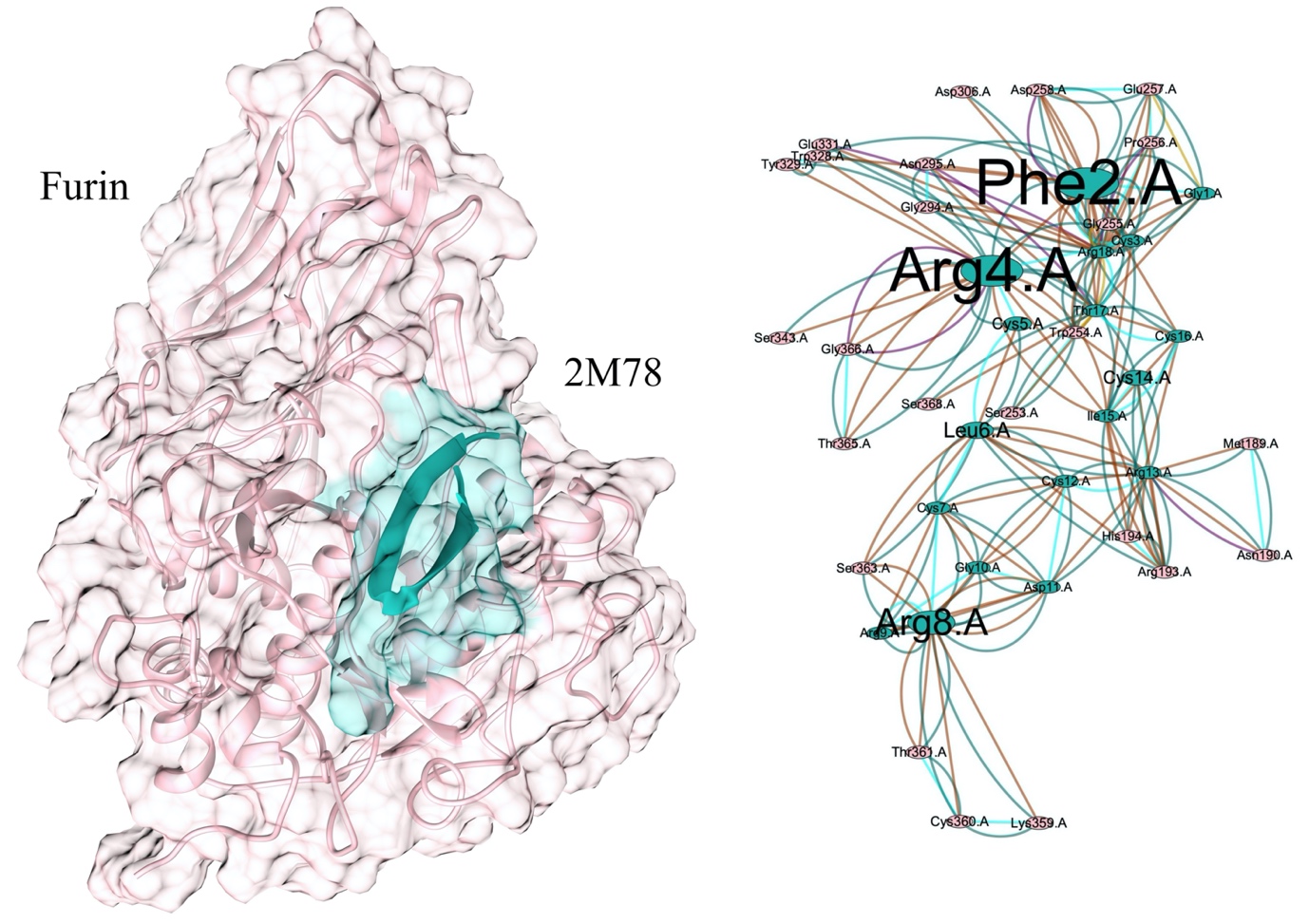

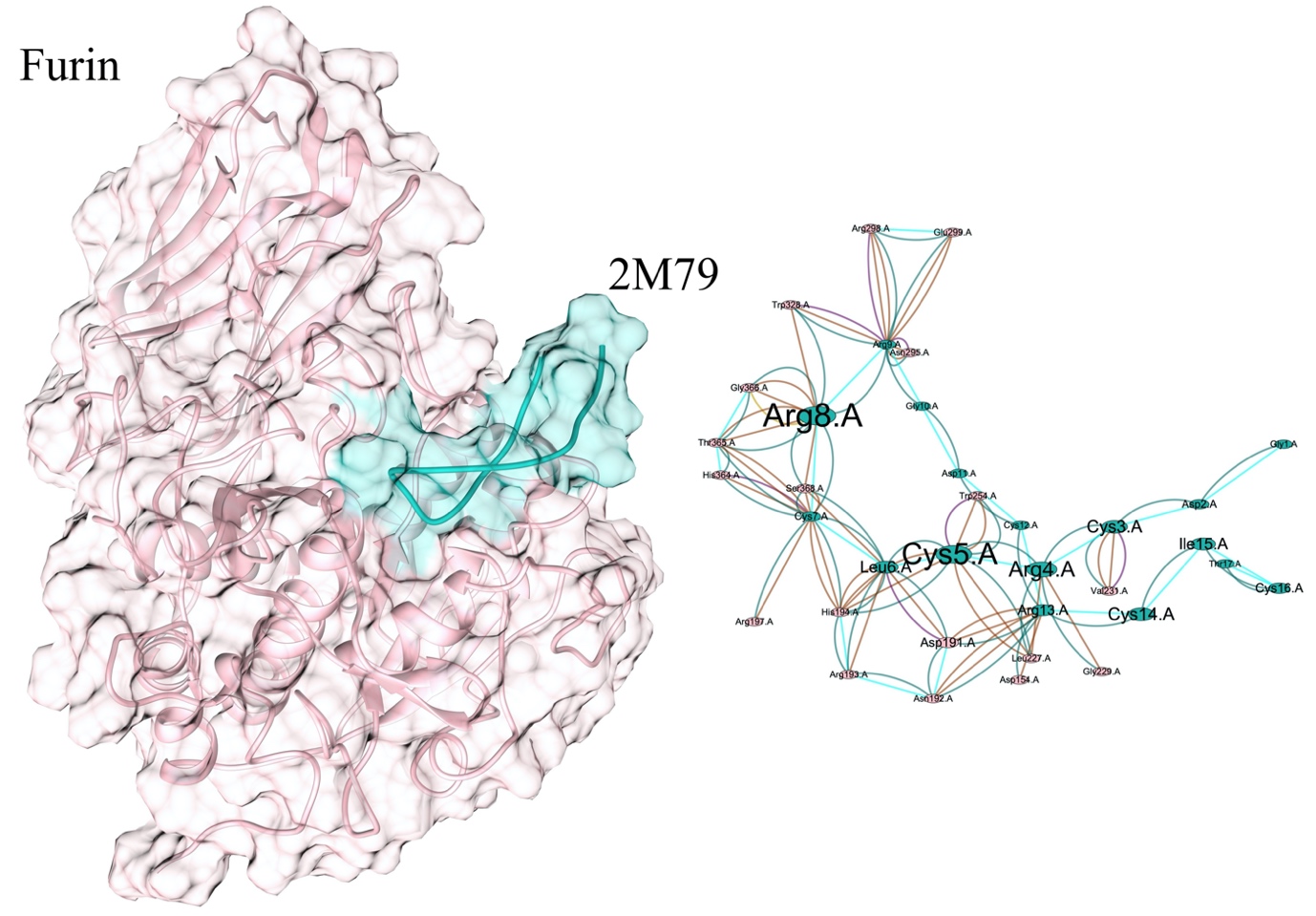

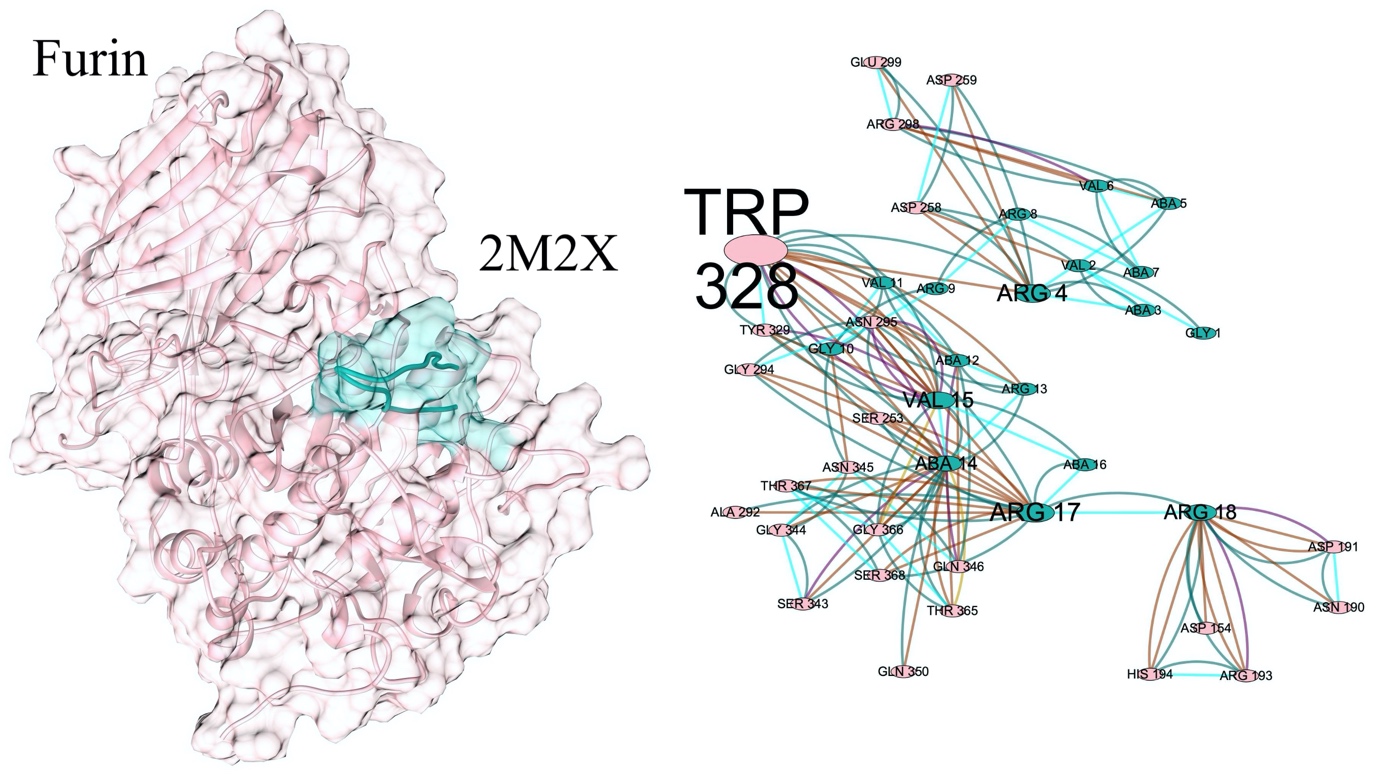

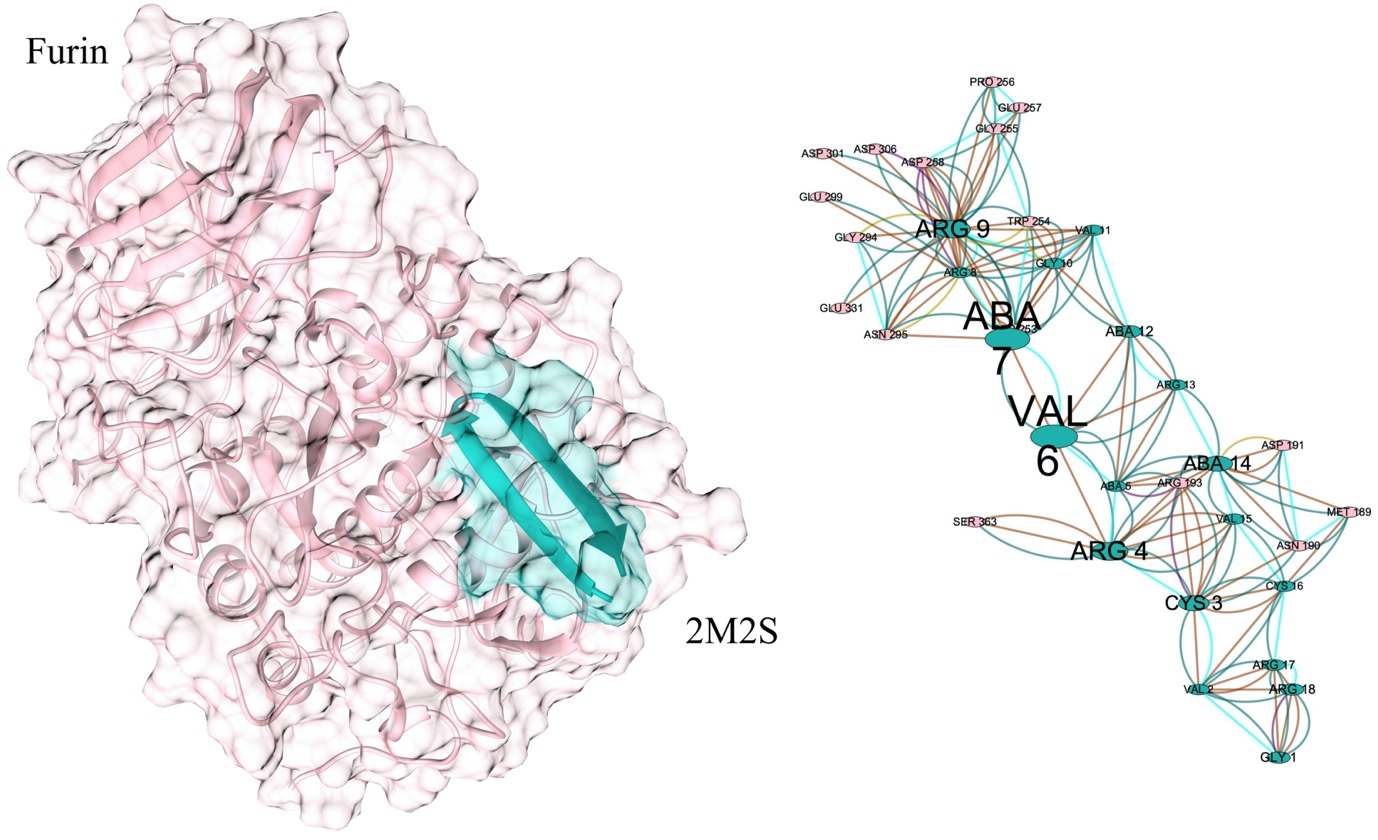

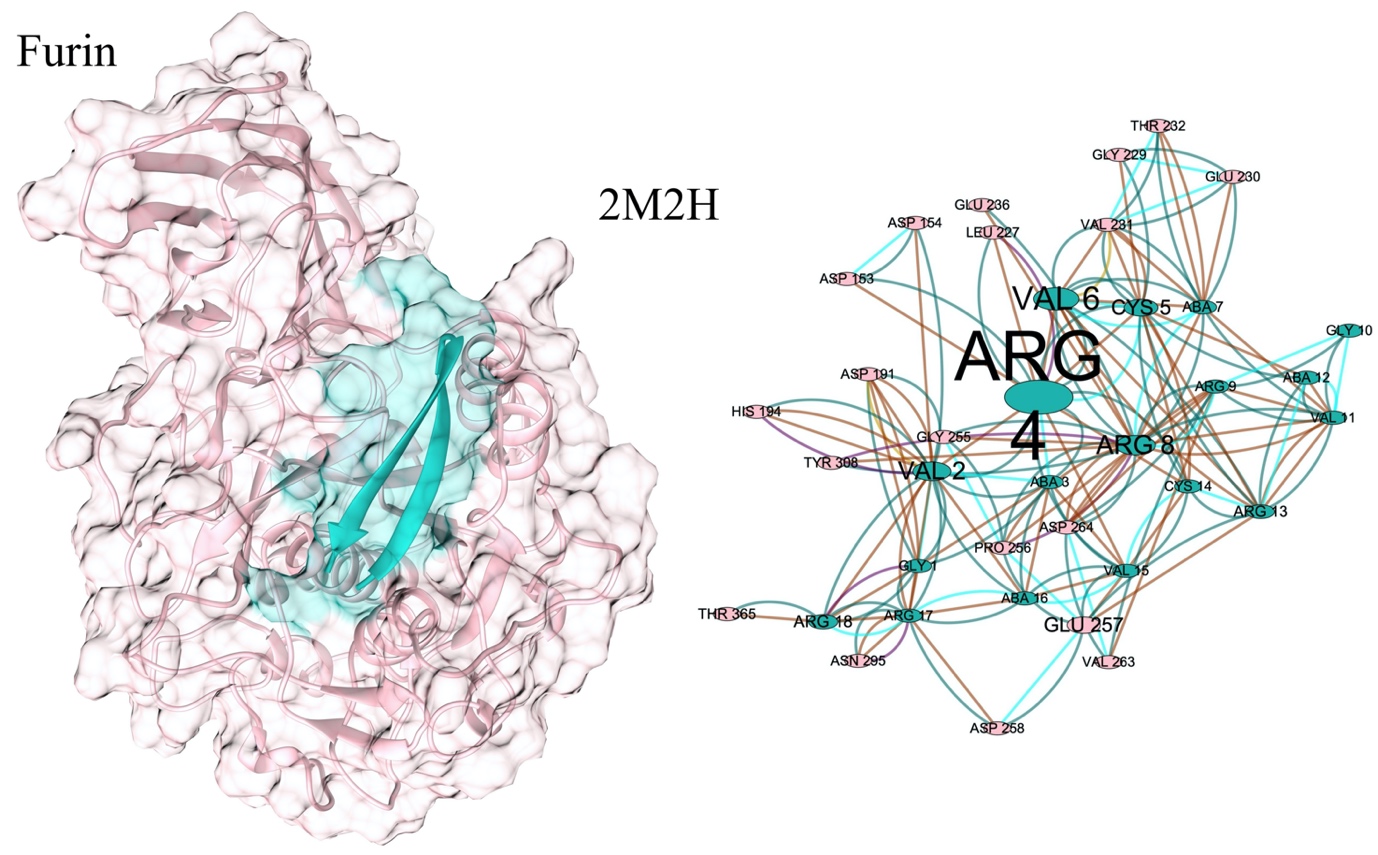

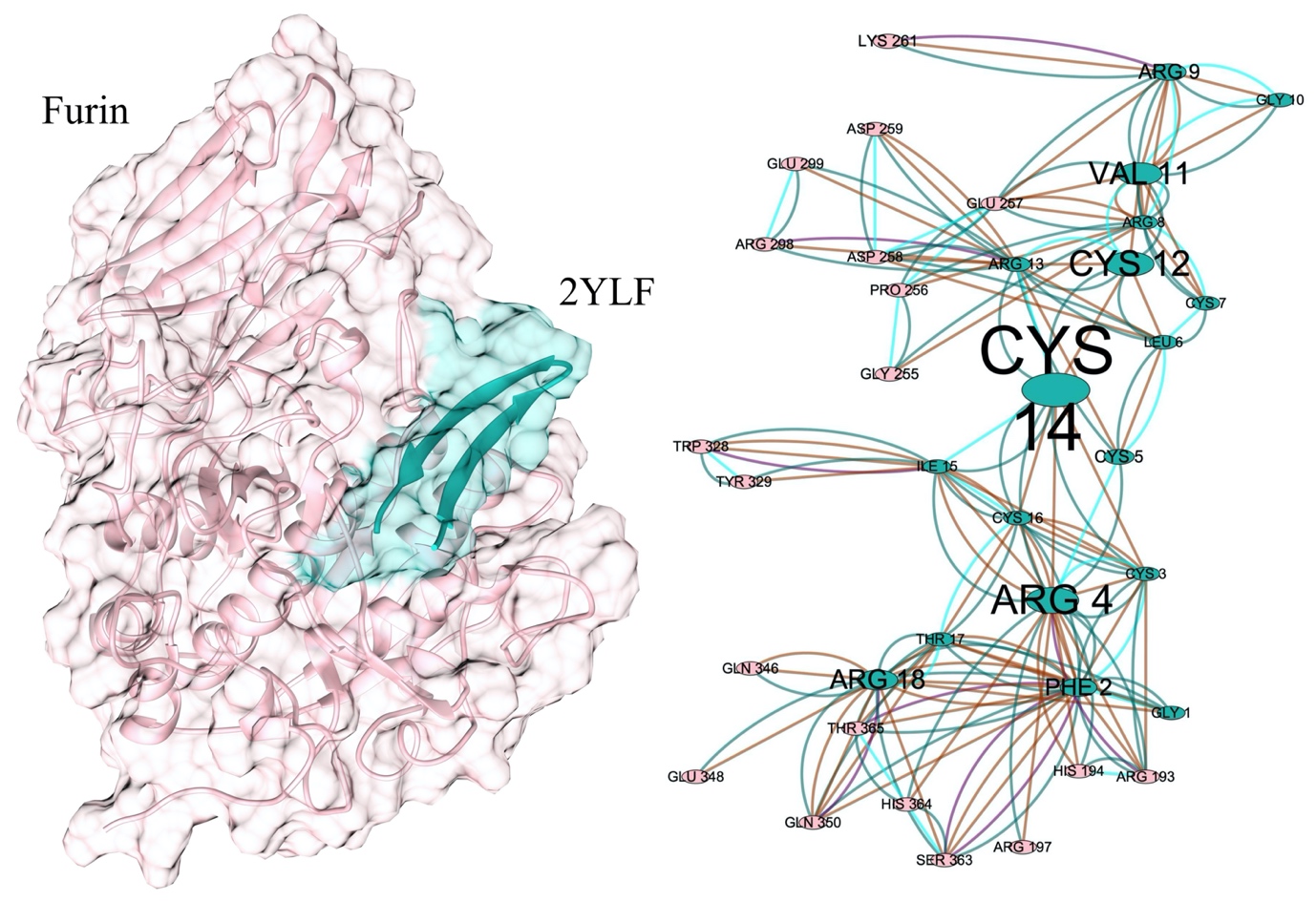

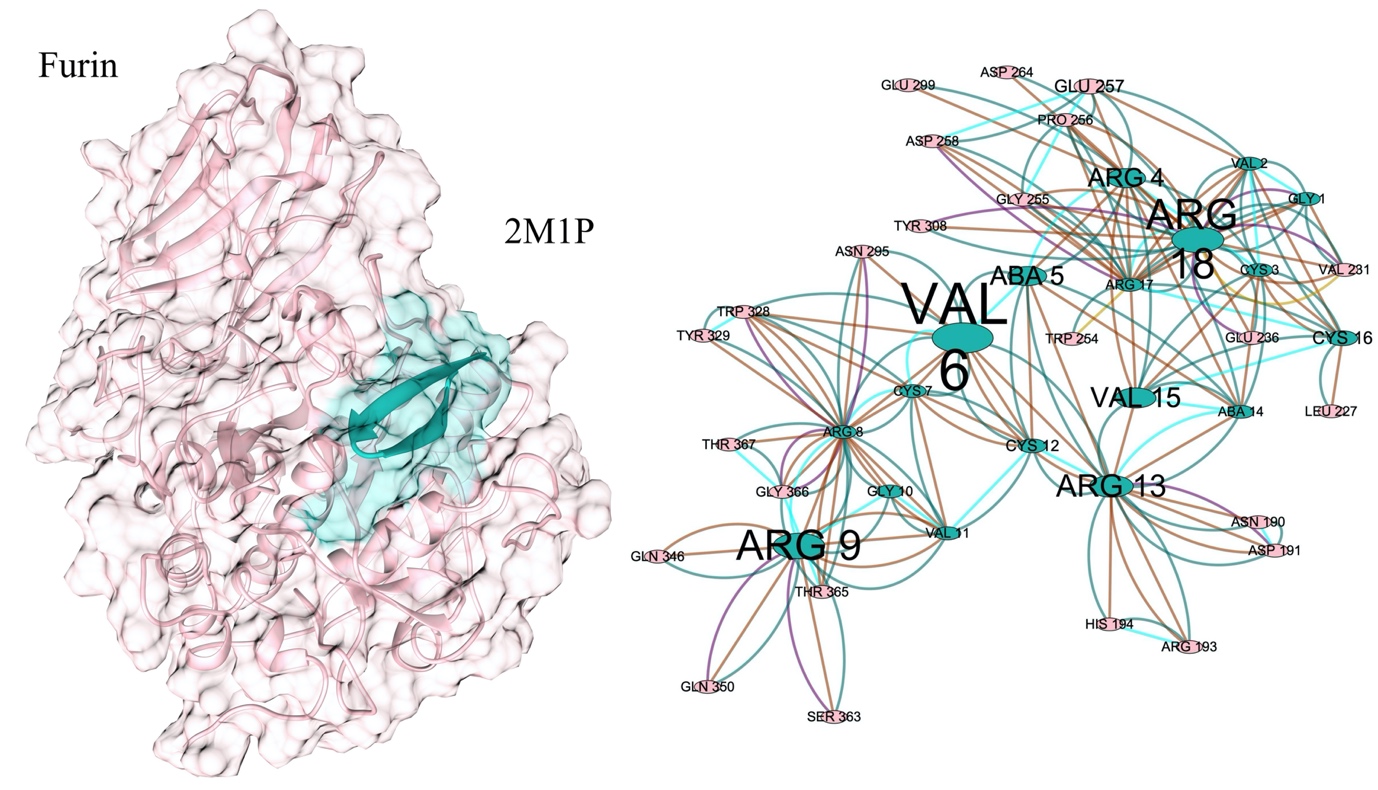


Figure S 4 A centrality analysis outlined the important residues involved in the peptide-furin complex. The Z-score values (significant Z-score: >2) of centrality reveals a pronounced role for arginine residues in each complex.
